# DNA Methyltransferase Inhibitor Zebularine Induces Human Cholangiocarcinoma Cell Death through Alteration of DNA Methylation Status

**DOI:** 10.1371/journal.pone.0120545

**Published:** 2015-03-23

**Authors:** Kazuaki Nakamura, Kazuhiko Nakabayashi, Kyaw Htet Aung, Kazuko Aizawa, Naoko Hori, Junji Yamauchi, Kenichiro Hata, Akito Tanoue

**Affiliations:** 1 Department of Pharmacology, National Research Institute for Child Health and Development, Tokyo, Japan; 2 Department of Maternal-Fetal Biology, National Research Institute for Child Health and Development, Tokyo, Japan; University of Medicine, Greifswald, Germany, GERMANY

## Abstract

Cholangiocarcinoma (CCA) is a cancer arising from the neoplastic transformation of cholangiocytes. During tumorigenesis, tumor suppressor and cancer-related genes are commonly silenced by aberrant DNA methylation in their promoter regions. Zebularine (1-(β-D-ribofuranosyl)-1,2-dihydropyrimidin-2-one) acts as an inhibitor of DNA methylation and exhibits chemical stability and minimal cytotoxicity both *in vitro* and *in vivo*. In this study, we explore the effect and possible mechanism of action of zebularine on CCA cells. We demonstrate that zebularine exerts an antitumor effect on CCA cells. Zebularine treatment decreased the concentrations of DNA methyltransferase (DNMT) proteins, and DNMT1 knockdown led to apoptotic cell death in the CCA cell lines TFK-1 and HuCCT1. DNA methylation analysis demonstrated that zebularine induced DNA demethylation, and the GO Biological Process terms “hemophilic cell adhesion”, “regulation of transcription, DNA-dependent” and “Wnt signaling pathway” were found to be significantly enriched in association with demethylated genes. Furthermore, we observed that zebularine treatment decreased β-catenin protein levels in TFK-1 and HuCCT1 cells. These results suggest that zebularine alters DNA methylation status, and that some aspect of DNA demethylation by zebularine induces suppression of the Wnt signaling pathway, which leads to apoptotic cell death in CCA. We previously reported a novel mechanism of zebularine-induced cell growth arrest and apoptosis in hepatocellular carcinoma via a DNA methylation-independent pathway. Together, our present and previous studies indicate that zebularine could function as both a DNMT inhibitor and a non-DNMT inhibitor reagent, and that, while the optimal usage of zebularine may depend on cancer type, zebularine may be useful for chemotherapy against cancer.

## Introduction

Cholangiocarcinoma (CCA) is a cancer arising from the neoplastic transformation of cholangiocytes. Several epidemiological studies show that the incidence and mortality rates of CCA have been increasing worldwide over the past few decades. In Europe, approximately 50,000 new cases of primary liver cancer are diagnosed each year. Data from the Cancer Incidence in Five Continents survey indicate that approximately 20% of those cases are attributed to CCA [1]. Although surgical excision is considered the most effective therapeutic approach, the five-year survival rate is still lower than 5%. CCA is characterized by late diagnosis, poor prognosis and lack of response to both chemotherapy and radiation therapy. In addition, the only effective treatment, surgical excision, is frequently not applicable because of delayed diagnosis [2]. Thus alternative chemotherapeutic strategies must be developed for the treatment of CCA [3, 4].

In recent years, much has been learned about epigenetic change, which has been confirmed as an important mechanism in multiple tumors. Epigenetic change is defined as any heritable modification of the genome that is not accompanied by a change in the DNA sequence [5]. Methylation is restricted to the CpG dinucleotides, which are largely depleted from the genomes except in short genomic regions called CpG islands, which commonly represent promoters [6]. Aberrant promoter methylation is initiated at about 1% of all CpG islands, and as many as 10% of all CpG islands become methylated during the multistep process of tumorigenesis [7]. The hypermethylation of promoter CpG islands can result in gene silencing, an alternative mechanism of gene inactivation that contributes to the formation of tumors including CCA [8]. Given that aberrant methylation is a major event in the early and late stages of tumorigenesis [9, 10], this process may represent a critical target for cancer risk assessment, treatment, and chemoprevention [11].

DNA methylation is specifically mediated by the action of DNA methyltransferase (DNMT) enzymes [12], which include DNMT1, DNMT3a, and DNMT3b [13]. DNMT1 has *de novo* as well as maintenance methyltransferase activity, and DNMT3a and DNMT3b are potent *de novo* methyltransferases [14]. Overexpression of DNMT has been reported to be involved in tumorigenesis [15] and has been suggested as a prognostic factor in diffuse large B-cell lymphomas [16]. Therefore, it has been proposed that the inhibition of DNMT activity can strongly reduce the formation of tumors [17]. Epigenetic changes such as DNA methylation are pharmacologically reversible. Thus far, three DNMT-inhibiting cytosine nucleoside analogs (5’-azacitidine, decitabine, and zebularine) have been studied as potential anticancer drugs [18–20]. Decitabine and 5’-azacitidine are widely used in the treatment of patients with various cancers, such as myelodysplastic syndromes (MDS) and acute myeloid leukemia (AML) [21, 22]. In CCA, treatment with decitabine decreased cell proliferation, growth in soft agar, and methylcytosine content of malignant cholangiocytes [23]. Although decitabine and 5’-azacitidine are effective in treating various cancers [21, 22], the formation of irreversible covalent adducts with DNA may cause long-term side effects, including DNA mutagenesis, a potential cause of tumor recurrence. In addition, these drugs have short-term side effects. The most common toxicity is myelosuppression, mainly displaying as neutropenia and thrombocytopenia [24]. Furthermore, decitabine and 5’-azacitidine have been demonstrated to cause both DNA hypomethylation and DNA damage, albeit at lower concentrations [25].

Zebularine is a second-generation, highly stable hydrophilic inhibitor of DNA methylation with oral bioavailability that preferentially targets cancer cells [11], as demonstrated in bladder, prostate, lung, colon, and pancreatic carcinoma cell lines [26]. It acts primarily as a trap for DNMT proteins by forming tight covalent complexes between DNMT proteins and zebularine-substitute DNA [27]. Zebularine is also a cytidine analog that was originally developed as a cytidine deaminase inhibitor. It exhibits low toxicity in mice, even after prolonged administration [28–30]. Zebularine exerts antitumor activity on cells of the hepatocellular carcinoma cell line HepG2 by inhibiting cell proliferation and inducing apoptosis [31]. Little is known, however, about the anticancer effect and possible mechanism of action of zebularine on CCA.

In the present study, we investigated the effect of zebularine against CCA, and demonstrated that zebularine exhibited anticancer activity against CCA. Zebularine induced apoptosis of CCA cells via DNMT1 inhibition. Zebularine altered DNA methylation status and demethylated many CpG sites including “hemophilic cell adhesion”, “regulation of transcription, DNA-dependent” and “Wnt signaling pathway” genes. In addition, zebularine decreased β-catenin protein levels in CCA cells. These results suggest that zebularine affects DNA methylation status and the expression patterns of Wnt signaling pathway-related genes, thus inhibiting the Wnt signaling pathway and inducing apoptosis in CCA.

## Materials and Methods

### Cell culture

TFK-1 [32] (RCB2537) and HuCCT1 [33] (RCB1960) were provided by the RIKEN BRC through the National Bio-Resource Project of MEXT, Japan. KKU-100 (JCRB1568), KKU-M156 (JCRB1561) [34] and KKU-M213 [35] (JCRB1557) were provided by the JCRB cell bank at the National Institute of Biomedical Innovation, Japan. TFK-1, HuCCT1 and KKU-M213 were maintained at 37°C under an atmosphere of 95% air and 5% CO_2_ in RPMI1640 containing 10% fetal bovine serum (FBS), 100 U/ml penicillin, and 100 μg/ml streptomycin. KKU-100 and KKU-M156 were maintained at 37°C under an atmosphere of 95% air and 5% CO_2_ in DMEM containing 10% fetal bovine serum (FBS), 100 U/ml penicillin, and 100 μg/ml streptomycin. Cells were immersed in a culture medium containing the indicated zebularine concentrations. Zebularine (Wako Pure Chemical Industries, Osaka, Japan) was dissolved in distilled water as a stock solution.

### Cell viability assay

Cell viabilities were determined by means of WST assay or CellTiter-Glo Luminescent Cell Viability Assay. The WST assay was performed using a Cell Counting Kit-8 (Dojindo Laboratories, Kumamoto, Japan) according to the manufacturer’s instructions. The CellTiter-Glo Luminescent Cell Viability Assay kit was purchased from Promega KK (Tokyo, Japan). Cell cultures exposed to 0 μM zebularine or 0 nM siRNA (control) were considered to be 100% viable. The cell viability of each treated sample was presented as a percentage of the viability of cultures treated with control. All samples were run at least three times in the same assay.

### Immunoblotting

Cells were lysed in lysis buffer (20 mM HEPES–NaOH pH 7.5, 150 mM NaCl, 1% NP-40, 1.5 mM MgCl2, 1 mM EGTA, 1 μg/ml leupeptin, 1 mM PMSF, and 1 mM Na_3_VO_4_) and stored at -80°C until use. After centrifugation, aliquots of the supernatants were subjected to sodium dodecyl sulfate polyacrylamide gel electrophoresis (SDS-PAGE). The electrophoretically separated proteins were transferred to polyvinylidene fluoride (PVDF) membranes, blocked, and immunoblotted with anti-DNMT1 (D63A6, #5032, Cell Signaling Technology Japan, Tokyo, Japan), DNMT3a (sc-20703), DNMT3b (sc-81252), β-catenin (sc-1496) (Santa Cruz Biotechnology, Santa Cruz, CA, USA), or glyceraldehyde 3-phosphate dehydrogenase (GAPDH) (#MAB374, Millipore, Temecula, CA, USA) antibodies (each 1:1000 dilution), and then with peroxidase-conjugated secondary antibodies (NA931 or NA940, each 1:10000 dilution, GE Healthcare Japan, Tokyo, Japan, or sc-2020, 1:5000 dilution, Santa Cruz Biotechnology). The bound antibodies were detected using the ECL system (GE Healthcare, Tokyo, Japan).

### siRNA and transfection

The siRNA against DNMT1 (HSC.RNAI.N001130823.12.1; siDNMT1#1 and HSC.RNAI.N001130823.12.3; siDNMT1#2) and negative control (DS NC1) were purchased from Integrated DNA Technologies (Coralville, IA, USA). Transient siRNA transfection in TFK-1 and HuCCT1 cells was performed according to the Lipofectamine RNAiMAX (Invitrogen, Life Technologies Japan, Tokyo, Japan) method.

### Apoptotic/necrotic/healthy cell detection assay

Apoptotic, necrotic, and healthy cells were detected using an apoptotic/necrotic/healthy cell detection kit (PromoCell, Heidelberg, Germany). After 72 h of incubation with zebularine, cells were incubated for 15 min with FITC-Annexin V, ethidium homodimer I (EthD-I) and Hoechst 33342 (included in the kit). The assay was performed according to the manufacturer’s instructions. Fluorescence images were photographed and analyzed using IN CELL Analyzer 2200 (GE Healthcare). All samples were run at least three times in the same assay.

### Apoptosis analysis

Quantification of apoptotic cells was performed using a Cell Death Detection ELISA^PLUS^ (Roche Diagnostics, Tokyo, Japan) according to the manufacturer’s instructions. The assay allows the specific determination of mono- and oligonucleosomes in the cytoplasmatic fraction of cell lysates. The enrichment of mono- and oligonucleosomes in the cytoplasm occurs due to DNA degradation in the apoptotic cells before plasma membrane breakdown. After 24 h or 72 h of incubation with zebularine, or 72h of siRNA treatment, cells were lysed with a lysis buffer (included in the kit). Absorbance values were measured at 405 nm using a microplate reader (ARVO, PerkinElmer Japan, Kanagawa, Japan). The apoptotic ratio of each drug-treated sample was presented as a fold-change from the apoptotic ratio of cultures treated with control. All samples were run at least three times in the same assay.

### 5-bromo-2’-deoxy-uridine (BrdU) incorporation assay

Cellular DNA synthesis rates were determined by measuring BrdU incorporation with the commercial Cell Proliferation ELISA System (Roche Diagnostics). After 24 h of incubation with zebularine, cells were incubated for 2 h with a BrdU labeling solution (included in the kit) containing 10 μM BrdU. The assay was performed according to the manufacturer’s instructions. Absorbance values were measured at 405 nm using a microplate reader. The BrdU incorporation of each drug-treated sample was presented as a fold-change of the BrdU incorporation of cultures treated with 0 μM zebularine. All samples were run at least three times in the same assay.

### Caspase assays

After 72 h of incubation with zebularine, Caspase-3/7, -8, and -9 activities were assayed with Caspase-Glo Assays (Promega KK) according to the manufacturer’s standard cell-based assay protocols. The luminescence of each sample was measured using a plate-reading luminometer. Comparison of the luminescence from a treated sample with that from a control sample enabled determination of the relative increase in caspase activity. All samples were run at least three times in the same assay.

### Illumina Infinium HumanMethylation450 BeadChip analysis

Genomic DNA was extracted from three independent cell culture batches of zebularine (1000 μM)-treated and control TFK-1 or HuCCT1 cells after 72h zebularine treatment. Genomic DNA (1000 ng) was bisulfite-treated and purified using the EpiTect Bisulfite Plus Kit (QIAGEN K.K., Tokyo, Japan). Three hundred nanograms of bisulfite-treated DNA were hybridized to the Illumina Infinium HumanMethylation450 BeadChip using Illumina-supplied reagents and protocols. Both the CpG loci included on this array and the technologies behind the platform have been described previously [36]. GenomeStudio software (Illumina) was used to calculate the methylation level at each CpG site as a beta value (β = intensity of the methylated allele [M] / [intensity of the unmethylated allele (U) + intensity of the methylated allele (M) + 100]) (36).

### DNA damage analysis

After 72 h of incubation with zebularine, DNA damage was assayed with a DNA Damage Quantification Kit (Dojindo Laboratories) according to the manufacturer’s instructions. The assay allows determination of abasic sites (AP sites) in DNA samples. Oxidative attack by hydroxyl radicals on the deoxyribose moiety will lead to the release of free bases from DNA, generating strand breaks with various sugar modifications and simple AP sites. Genomic DNA were extracted from three independent cell culture batches. Absorbance values were measured at 650 nm using a microplate reader (ARVO). The number of AP sites in the genomic DNA was determined using the calibration curve.

### Immunohistochemistry

The cholangiocarcinoma and normal tissue sections (Tissue Microarray; Human Cholangio Carcinoma) were purchased from provitro AG (Berlin, Germany). Provitro AG obtained human samples from partnering hospitals in accordance with an approved partnering hospital review board protocol. The sections were boiled with 10 mM citrate buffer (pH 6.0) for 10 min by microwave. The sections were treated with 0.3% hydrogen peroxide for 10 min and blocked with PBS containing 5% FBS and 0.1% Triton X-100. They were then incubated for 1 h at room temperature with an anti-DNMT1 (10 μg/ml, ab19905, abcam, Tokyo, Japan). The sections were then incubated with horseradish peroxidase conjugated secondary antibody (Envision; Dako Japan, Tokyo, Japan) for 30 min at room temperature and visualized using a Liquid DAB Substrate Chromogen System (Dako).

### Statistics

All experiments were performed at least three times. Values are expressed as means ± standard error of the mean (SEM). Statistical analyses were performed using an unpaired Student’s *t*-test or two-way analysis of variance (ANOVA) followed by Fisher’s protected least significant difference as a post-hoc test. *p* < 0.05 was considered to indicate statistical significance.

## Results

### The effects of zebularine on CCA viability

**Fig 1:**
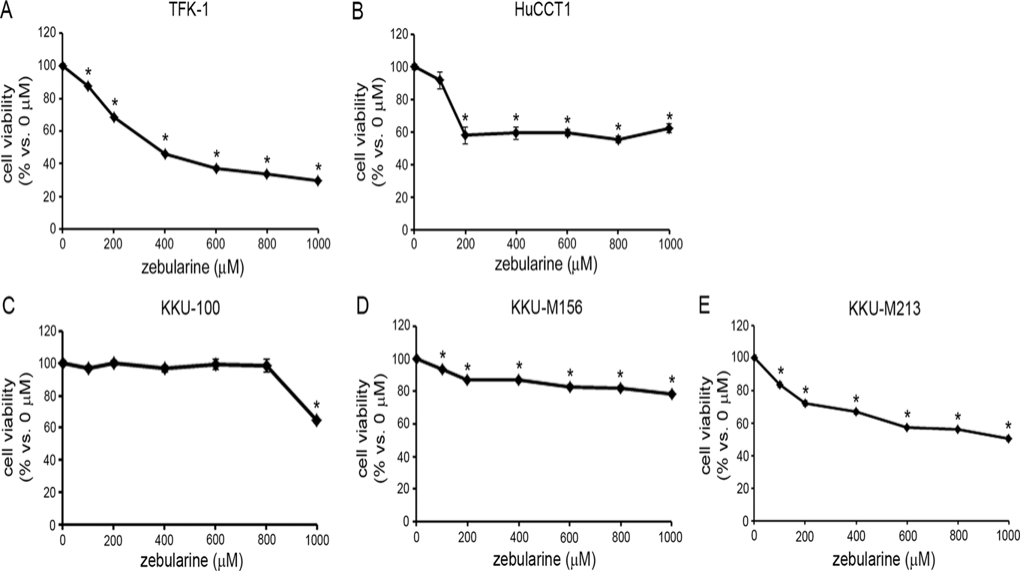
Effect of zebularine on cell viability in CCA cell lines. TFK-1 (A), HuCCT1 (B), KKU-100 (C), KKU-M156 (D) and KKU-M213 (E) cells were treated with zebularine at indicated concentrations for 72 h. Cell growth was measured by WST assay. Data are the means ± SEM of results from at least three independent experiments. *p < 0.05, compared to 0 μM.

**Fig 2:**
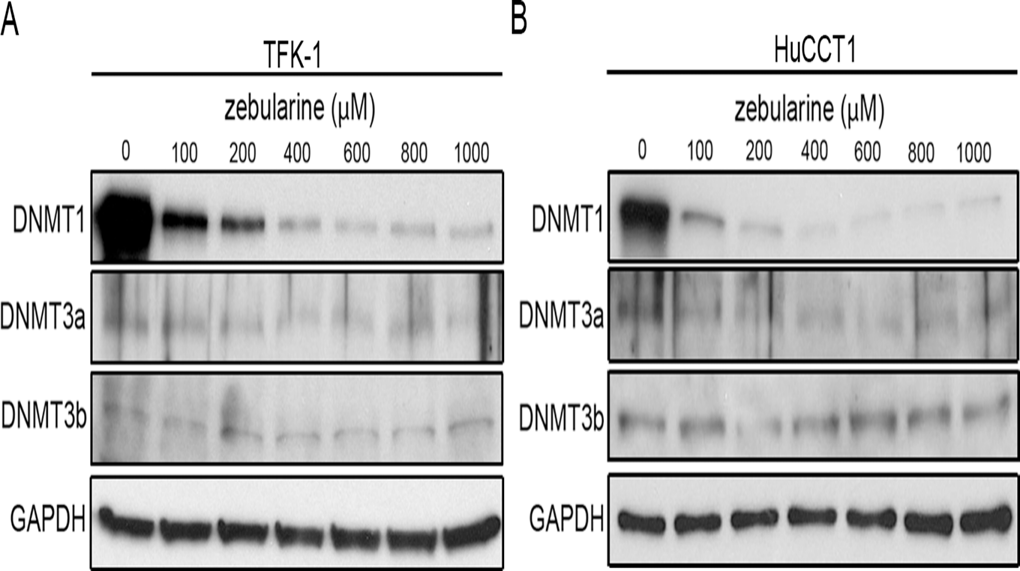
Effect of zebularine on DNMT expression in CCA cell lines. The protein levels of DNMT1, DNMT3a, and DNMT3b in TFK-1 (A) and HuCCT1 (B) cells after zebularine treatment for 72 h at different concentrations. After treatment, the cells were harvested and western blot analysis was performed to detect the protein levels of DNMT1, DNMT3a, and DNMT3b. GAPDH was used as a loading control.

In order to investigate the effect of zebularine on CCA viability, we performed a WST Assay and a CellTiter-Glo Luminescent Cell Viability assay after exposing five representative CCA cell lines to zebularine. The WST assay indicated that zebularine affected the viabilities of five cell lines: exposure of cells to zebularine for 72 h resulted in a decrease in cell viability in all five CCA cell lines examined in this study (Fig. 1A-1F). There were some differences in sensitivity among these CCA cell lines: TFK-1, HuCCT1 and KKU-M213 had relatively high sensitivity to zebularine compared to KKU-100 and KKU-M156. The CellTiter-Glo Luminescent Cell Viability assay likewise indicated that zebularine affected the viabilities of all five cell lines (S1 Fig.). Since the results of the WST assay and those of the CellTiter-Glo Luminescent Cell Viability assay were comparable, we used only the WST Assay for subsequent cell viability assays. Because of zebularine’s activity as a DNMT inhibitor in other model systems [31, 37, 38], its effect on the expression of DNMTs was examined in TFK-1 and HuCCT1 cells, two of the more sensitive CCA cell lines examined in this study. We measured the expression patterns of DNMT1, 3a and 3b in these cell lines, and found that zebularine treatment was mainly associated with dose-dependent depletion of DNMT1 (Fig. 2A, 2B). These results suggest that DNMT1 depletion resulting from zebularine treatment caused the observed reduction in cell viability in both TFK-1 and HuCCT1 cells.

### The effects of DNMT1 knock-down on CCA viability

**Fig 3:**
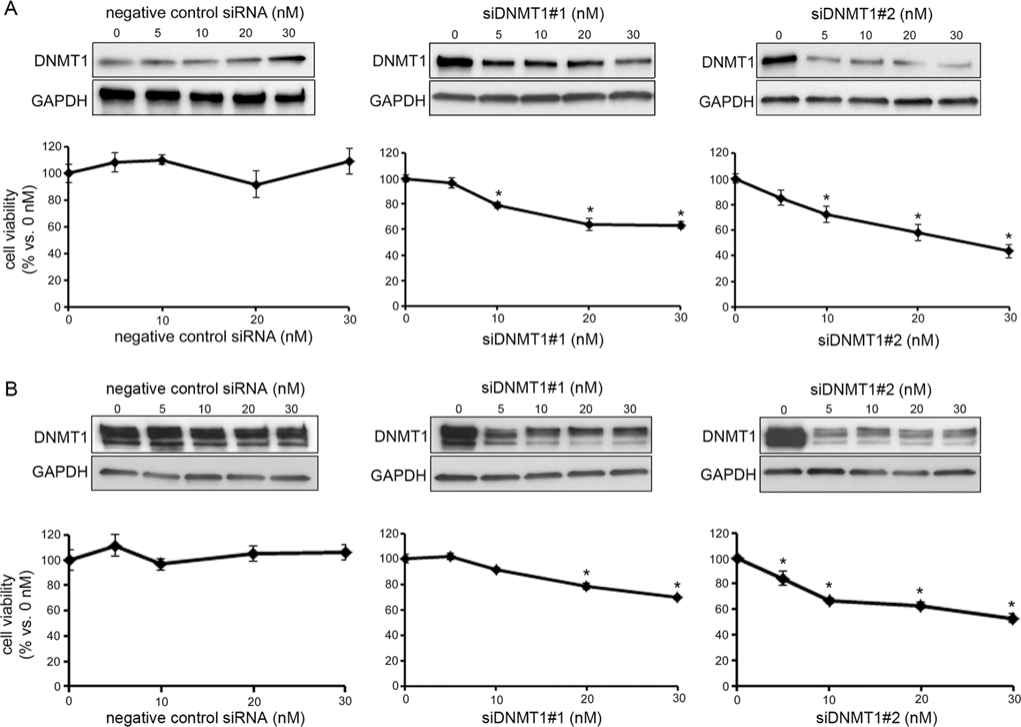
Effect of DNMT1 knockdown on cell viability in CCA cell lines. The forward transfection of control siRNA (control) as the negative control, siDNMT1#1 or siDNMT1#2 at indicated concentrations was performed; after 72 h of transfection, the protein levels of DNMT1, DNMT3a, DNMT3b and GAPDH were detected by western blot analysis, and cell growth was measured by WST assay in TFK-1 (A) and HuCCT1 (B). Data are the means ± SEM of results from at least three independent experiments. *p < 0.05, compared to control.

In order to examine whether the effect of zebularine on TFK-1 and HuCCT1 cell viability is due to DNMT1 depletion, we performed DNMT1 knock-down experiments. Transfection of siRNA against DNMT1 reduced DNMT1 protein in TFK-1 cells in an siRNA-dose-dependent manner, and simultaneously reduced TFK-1 cell viability in an siRNA-dose-dependent manner (Fig. 3A). Similarly, siRNA against DNMT1 reduced DNMT1 protein in HuCCT1 cells in an siRNA-dose-dependent manner and also reduced HuCCT1 cell viability (Fig. 3B). Negative control siRNA had no effect on DNMT1 protein level or cell viability in either TFK-1 or HuCCT1 cells. These results suggest that zebularine affects the cell viability of TFK-1 and HuCCT1 cells via DNMT1 depletion.

### Zebularine induces apoptosis in CCA

**Fig 4:**
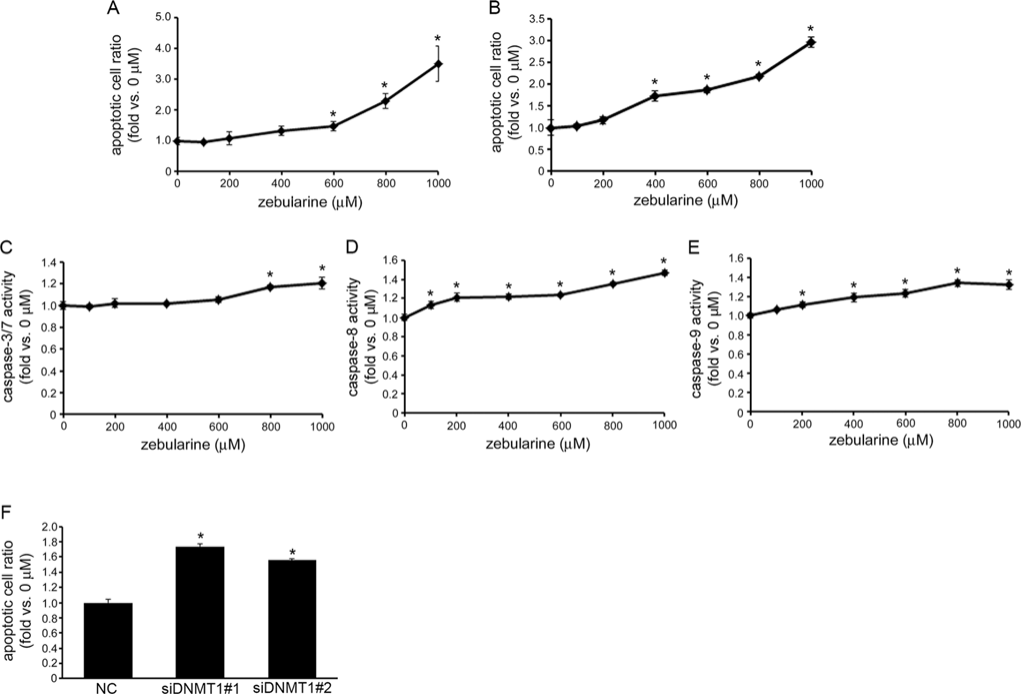
Effects of zebularine on apoptotic cell death in TFK-1 cells. TFK-1 cells were treated with zebularine at indicated concentrations for 72 h. Apoptosis was measured using apoptotic/necrotic/healthy cell detection assay (A) and Cell Death Detection ELISA assay (B). The data are expressed as fold-increases relative to the respective untreated samples. Caspase-3/7 (C), -8 (D), and -9 (E) activities were determined using Caspase-Glo Assays. The data are expressed as fold-increases relative to the respective untreated samples (RLU/60 min/mg protein). (F) Forward transfection of negative control siRNA (NC) as the control, siDNMT1#1 or siDNMT1#2 at 30 nM was performed, and apoptosis was measured by Cell Death Detection ELISA assay. Data are the means ± SEM of results from at least three independent experiments. *p < 0.05, compared to 0 μM.

To determine whether zebularine’s reduction of cell viability in TFK-1 and HuCCT1 cells is due to cell death, we measured necrotic and apoptotic cell death in TFK-1 and HuCCT1 cells exposed to zebularine. An apoptotic/necrotic/healthy cell detection assay revealed that, in TFK-1 cells exposed to zebularine for 72 h, zebularine did not induce necrotic cell death (data not shown); rather, it induced apoptotic cell death in a dose-dependent manner (Fig. 4A). In addition, we measured apoptotic cell death resulting from zebularine treatment in TFK-1 using a Cell Death Detection ELISA assay. This assay also indicated that exposure of cells to zebularine for 72 h resulted in an increase in the apoptotic ratio in TFK-1 cells (Fig. 4B). To investigate whether zebularine-induced apoptosis in TFK-1 cells was associated with the caspase family of proteins, the activity levels of caspase-3/7, -8, and -9 were examined after cells were exposed to zebularine for 72 h. As shown in Fig. 4C, the activity of caspase-3/7 in TFK-1 cells was significantly increased after exposure to zebularine, as were the activity levels of caspase-8 (Fig. 4D) and -9 (Fig. 4E). Furthermore, DNMT1 knock-down increased apoptotic cell death in TFK-1 cells (Fig. 4F).

**Fig 5:**
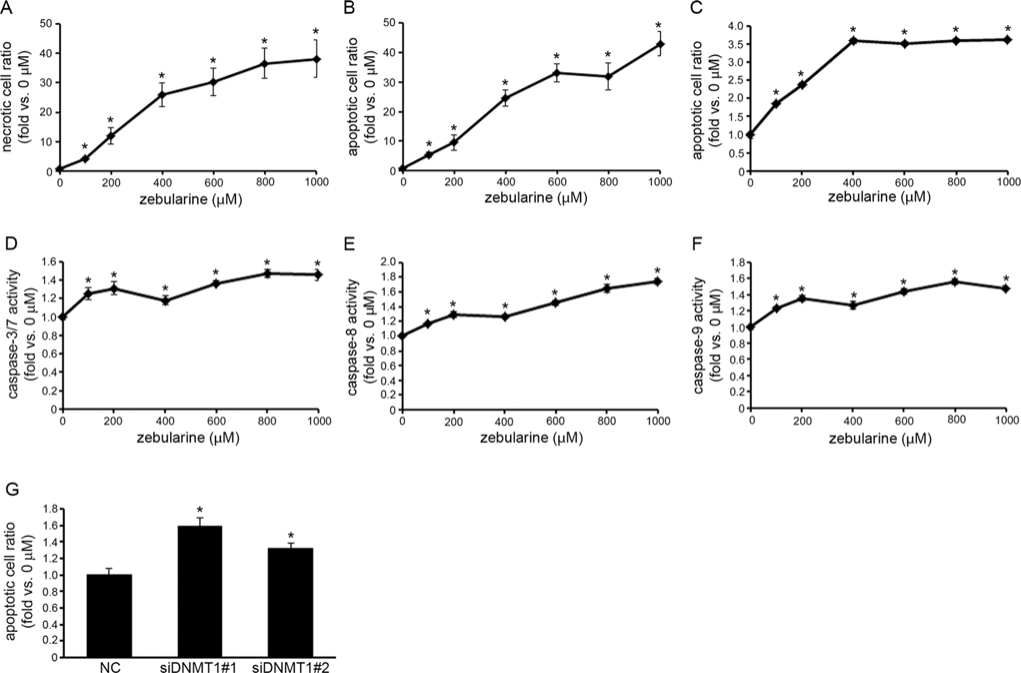
Effects of zebularine on apoptotic cell death in HuCCT1 cells. HuCCT1 cells were treated with zebularine at indicated concentrations for 72 h. Necrosis (A) and apoptosis (B) were measured using apoptotic/necrotic/healthy cell detection assay. (C) Apoptosis also was measured by Cell Death Detection ELISA assay (C). The data are expressed as fold-increases relative to the respective untreated samples. Caspase-3/7 (D), -8 (E), and -9 (F) activities were determined using Caspase-Glo Assays. The data are expressed as fold-increases relative to the respective untreated samples (RLU/60 min/mg protein). (G) Forward transfection of negative control siRNA (NC) as the control, siDNMT1#1 or siDNMT1#2 at 30 nM was performed, and apoptosis was measured by Cell Death Detection ELISA assay. Data are the means ± SEM of results from at least three independent experiments. *p < 0.05, compared to 0 μM.

Turning to HuCCT1 cells, an apoptotic/necrotic/healthy cell detection assay revealed that exposure to zebularine for 72 h induced necrotic cell death (Fig. 5A) and apoptotic cell death (Fig. 5B) in these cells as well. In addition, a Cell Death Detection ELISA assay indicated that exposure of HuCCT1 cells to zebularine for 72 h resulted in an increase in the apoptotic ratio (Fig. 5C). To investigate whether zebularine-induced apoptosis in HuCCT1 cells was associated with the caspase family of proteins, the activity levels of caspase-3/7, -8, and -9 were examined after zebularine treatment for 72 h. As shown in Fig. 5D, the activity of caspase-3/7 in HuCCT1 cells was significantly increased after zebularine treatment, as were the activity levels of caspase-8 (Fig. 5E) and -9 (Fig. 5F). Furthermore, DNMT1 knock-down increased apoptotic cell death in HuCCT1 cells (Fig. 5G).

These results indicate that apoptotic cell death was induced by treatment with zebularine through caspase activation via DNMT1 depletion in TFK-1 and HuCCT1 cells.

### Zebularine arrests HuCCT1 cell growth

**Fig 6:**
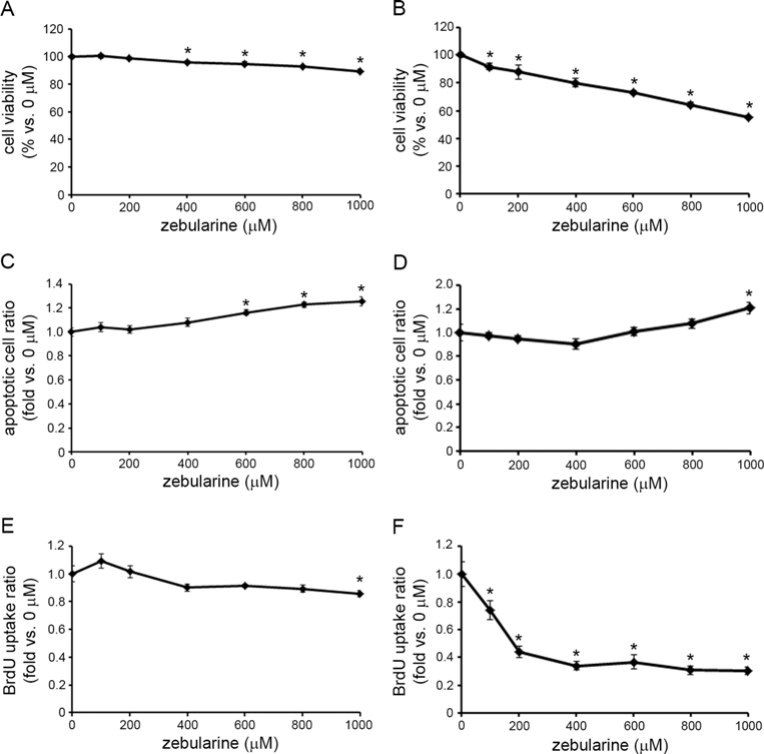
Effects of zebularine on cell growth in CCA cell lines. TFK-1 and HuCCT1 cells were treated with zebularine at indicated concentrations for 24 h. Cell growth was measured by WST assay in TFK-1 (A) and HuCCT1 (B) cells. Apoptosis was measured by Cell Death Detection ELISA assay in TFK-1 (C) and HuCCT1 (D) cells. Uptake of BrdU was measured by ELISA in TFK-1 (E) and HuCCT1 (F) cells. Data are the means ± SEM of results from at least three independent experiments. *p<0.05, compared to 0 μM.

To further determine whether zebularine could inhibit the proliferation of CCA, we conducted a BrdU incorporation assay in TFK-1 cells after zebularine treatment for 24 h. Although a WST assay indicated that zebularine could affect cell viability after 24 h (Fig. 6A), a BrdU incorporation assay showed that the uptake of BrdU by TFK-1 cells was reduced after 24 h of exposure to only 1000 μM zebularine (Fig. 6E). On the other hand, a cell death detection assay showed that apoptotic cell death was induced by zebularine treatment after 24 h (Fig. 6C). These results indicate that DNA replication in TFK-1 cells was not blocked by treatment with zebularine. We also conducted a BrdU incorporation assay in HuCCT1 cells after zebularine treatment for 24 h. A WST assay indicated that zebularine could affect cell viability after 24 h (Fig. 6B), while a simultaneous BrdU incorporation assay clearly showed that the uptake of BrdU by HuCCT1 cells was reduced after 24h exposure to zebularine (Fig. 6F). On the other hand, the cell death detection assay showed that apoptotic cell death was induced by 24 h of treatment with only 1000 μM zebularine (Fig. 6D). These results indicate that zebularine inhibits DNA replication in HuCCT1 cells.

### Zebularine alters DNA methylation status in TFK-1 and HuCCT1 cells

**Fig 7:**
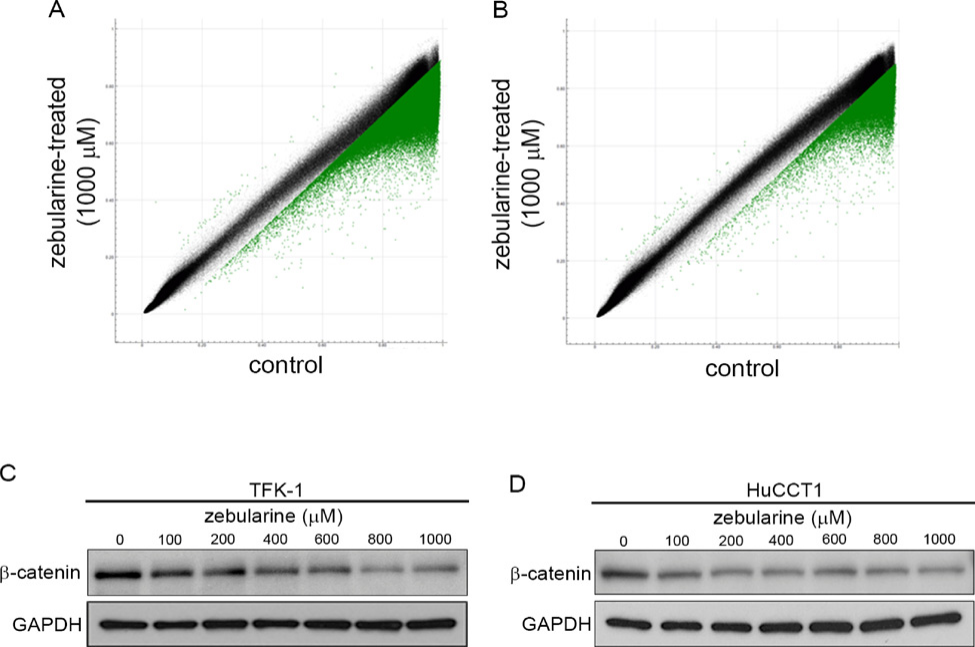
Effect of zebularine on DNA methylation and β-catenin expression in CCA cell lines. Scatter plot of the average beta values at 482,143 CpG sites for zebularine-treated (y-axis) and control (x-axis) TFK-1 (A) and HuCCT1 (B) cells (n = 3 for each group). Dots for CpG sites whose delta-beta value is >0.1 or <-0.1 are shown in green (59 [0.012%] hypermethylated and 151,482 [31.4%] hypomethylated CpG sites in TFK-1 cells and 70 [0.015%] hypermethylated and 47,438 [9.8%] hypomethylated CpG sites in HuCCT1 cells). The protein levels of β-catenin in TFK-1 (C) and HuCCT1 (D) cells after zebularine treatment for 72 h at different concentrations. After treatment, the cells were harvested and western blot analysis was performed to detect the protein levels of β-catenin. GAPDH was used as a loading control.

Given that zebularine decreases DNMT1 protein levels and induces apoptotic cell death, we next set out to determine whether the apoptosis and/or growth arrest induction caused by zebularine in TFK-1 and HuCCT1 cells results from alterations in DNA methylation. We obtained the genome-wide methylation profiles of zebularine-treated and -untreated (control) TFK-1 and HuCCT1 cells using an Illumina Infinium HumanMethylation450 BeadChip (GEO accession number GSE60446). Among 482,421 assays for CpG sites, 482,143 assays fulfilled our quality control criteria [detection *p* value <0.01 and no missing average beta value in both cell lines in the group comparison analysis using GenomeStudio V2011.1 (Illumina)], and were subjected to the following analysis. For each assay, the delta-beta value (= average of the beta values of three zebularine-treated samples—average of those of three controls) was calculated. As shown in Fig. 7, the methylation profiles of zebularine-treated and -untreated (control) cells differed in both TFK-1 (Fig. 7A) and HuCCT1 cells (Fig. 7B). In particular, the methylation statuses of highly methylated sites were affected by zebularine treatment. The numbers of CpG sites whose delta-beta values were >0.1 or <-0.1 were 151,541 (31.4%) and 47,508 (9.9%) in TFK1 and HuCCT1 cells, respectively. We annotated the functional and genomic features of notably hypomethylated (delta-beta < -0.2) regions using the Database for Annotation, Visualization, and Integrated Discovery (DAVID) v6.7 (http://david.abcc.ncifcrf.gov/). The numbers of CpG sites whose delta-beta values were <-0.2 were 21,214 (4.4%) and 5,297 (1.1%) in TFK1 and HuCCT1 cells, respectively. A total of 4,285 CpG sites from these two cell lines were commonly hypomethylated (delta-beta < -0.2) (S1 Table). Furthermore, 3,309 out of the 4,285 sites were found to be located in genic regions, while another 948 sites were located proximal to transcriptional start sites (referred to as “pTSS” hereafter; i.e., TSS1500, TSS200, the 5’ untranslated region [UTR], and the first exon categories). The results of gene ontology analyses for the 2,102 genes hosting the 3,309 CpG sites and the 782 genes hosting the 948 CpG sites are summarized in S2 Table and S3 Table, respectively. In both of the gene lists, the GO Biological Process terms “hemophilic cell adhesion” and “regulation of transcription, DNA-dependent” were found to be significantly enriched. The majority of the genes affiliated with the former term were protocadherin genes located at chromosome band 5q31. The genes affiliated with the latter term included many HOX cluster and homeobox genes encoding transcriptional regulators. It is also noteworthy that the genes categorized as belonging to the “Wnt signaling pathway” (S2 Fig.) in the Kyoto Encyclopedia of Genes and Genomes (KEGG) database were detected to be statistically significantly enriched among the 2,102 genes (but not among the 782 genes). Consistently, among the 31 genes that were categorized in the KEGG database as belonging to the “Wnt signaling pathway” and that also hosted hypomethylated CpG sites, only seven genes contained hypomethylated CpG sites at their pTSS regions (S4 Table). It has been reported that global alterations of DNA methylation in CCA target the Wnt signaling pathway, and that promoter silencing of Wnt signaling-related genes such as *SOX17*, *WNT3A*, *DKK2*, *SFRP1*, *SFRP2*, and *SFRP4* is reversed upon treatment of CCA cell lines with 5-aza-2’ deoxycytidine [39]. Among these six Wnt signaling-related genes, the promoter regions of *SOX17*, *DKK2*, *SFRP1*, and *SFRP2* were found to be hypomethylated after zebularine treatment in TFK1 and/or HuCCT1 cell lines (S1 Table and S3 Fig.). We visualized the beta and delta-beta values of TFK1 and HuCCT1 cell lines upon zebularine treatment together with the beta values of several human normal tissues (from GEO accession numbers GSE52578 and GSE30870) for the protocadherin gene cluster, *HOXA* gene cluster, homeobox genes (*IRX2* and *TLX3* as examples), and Wnt signaling-related genes (S3 Fig.) using Integrative Genomics Viewer (IGV, www.broadinstitute.org/igv/home). The majority of HOX cluster, homeobox and Wnt signaling-related genes that were found to be hypomethylated in TFK1 and/or HuCCT1 cell lines upon zebularine treatment were unmethylated at their promoter regions in normal tissues, and highly methylated in these CCA cell lines. Therefore, treatment of these CCA cell lines with zebularine is likely to partially reactivate any genes that were silenced during the carcinogenesis processes by demethylating their promoter regions, which may cause cancerous cells to commit to apoptotic cell death. These results suggest that zebularine reverses the promoter hypermethylation of protocadherin, transcriptional regulator, and Wnt signaling-related genes in CCA cell lines.

In order to confirm that zebularine actually affects the Wnt signaling pathway, we examined β-catenin protein levels after zebularine treatment. Zebularine treatment decreased β-catenin levels in a dose-dependent manner in TFK-1 and HuCCT1 cells (Fig. 7C, 7D), indicating that zebularine inhibits the Wnt signaling pathway in TFK-1 and HuCCT1 cells.

### High-concentration exposure to zebularine increases DNA damage in TFK-1 cells

**Fig 8:**
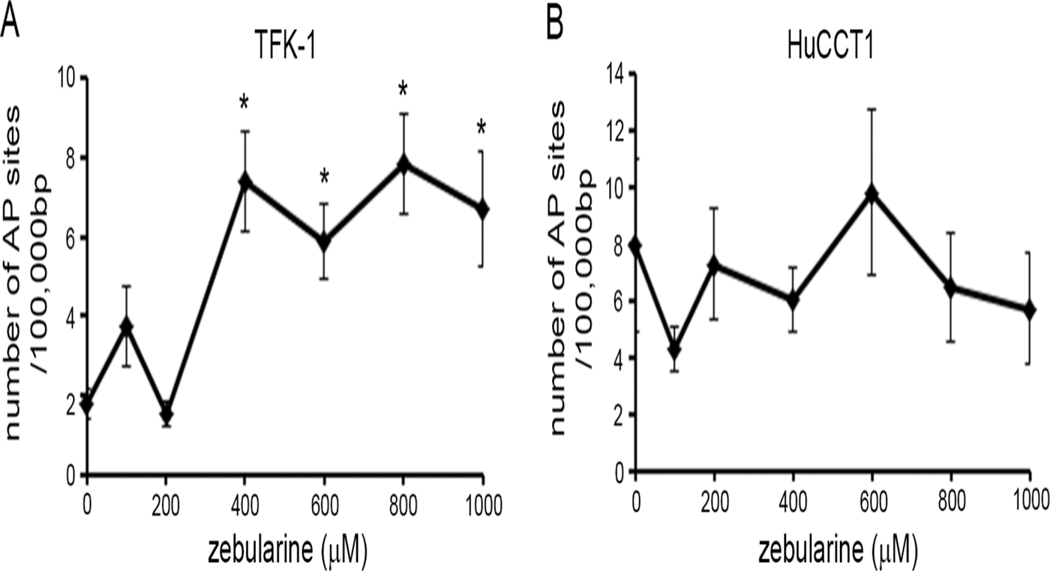
Effect of zebularine on DNA damage in CCA cell lines. The DNA damage in TFK-1 (A) and HuCCT1 (B) cells after zebularine treatment for 72 h at different concentrations. After treatment, DNA samples were extracted and DNA damage assay was performed. Data are the means ± SEM of results from at least three independent experiments. *p<0.05, compared to 0 μM.

Previous reports have shown that zebularine can induce DNA damage [40, 41]. In order to determine whether zebularine induces DNA damage in TFK-1 and HuCCT1 cells, we examined these cell lines for DNA damage after zebularine treatment. Zebularine treatment did not induce DNA damage in HuCCT1 cells in this study (Fig. 8B). In TFK-1 cells, on the other hand, high-concentration exposure to zebularine did increase DNA damage (Fig. 8A). These results indicate that the anticancer effect of zebularine does not depend on induction of DNA damage in HuCCT1 cells, but that it does partially depend on DNA damage in TFK-1 cells exposed to high concentrations of zebularine.

### DNMT1 is upregulated in human cholangiocarcinoma tissue

**Fig 9:**
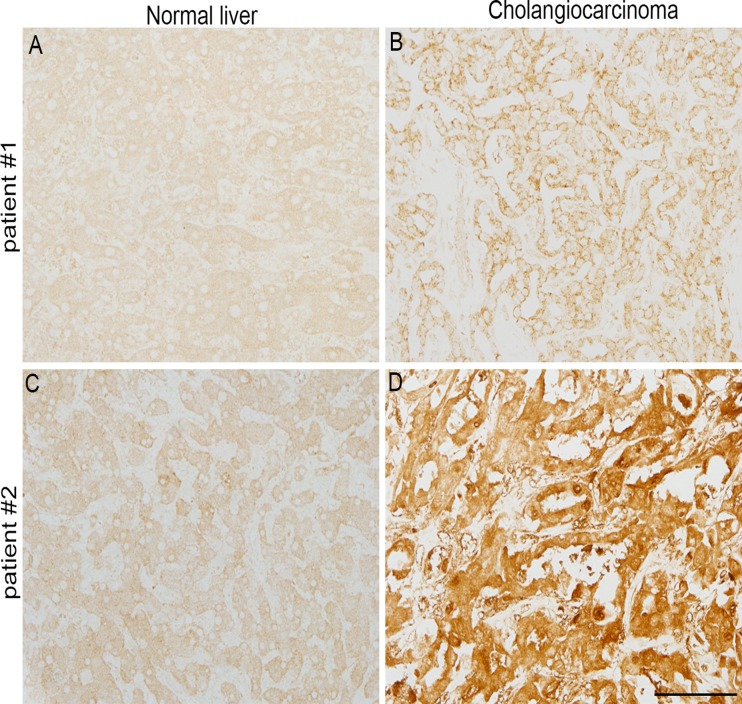
DNMT1 is upregulated in CCA tissues. DNMT1 immunoreactivities in human normal liver (A, C) and CCA tissues (B, D). A and B are derived from a single patient (patient #1; male, 74 years old), and C and D are derived from another single patient (patient #2; female, 67 years old). The immunohistochemical analyses were performed for DNMT1 protein in paraffin-embedded sections. Bar; 100 μm.

The expression levels of DNMTs are reportedly elevated in cancers of the colon [42], prostate [43], breast [44], and liver [45] and in leukemia [46]. These reports suggest that DNMT genes are upregulated in CCA tissues as well as in other cancers. Thus, we examined the expression of DNMT1 in CCA tissues. Immunohistochemical analysis revealed that immunoreactivities for DNMT1 were increased in CCA compared with normal tissues (Fig. 9), suggesting that upregulation of DNMT1 results in the development of CCA and that inhibition of DNMT1 is effective therapy against it.

## Discussion

Zebularine is a DNMT inhibitor with demonstrated antitumor effects against a range of cancer types [26]. In this study, we investigated the efficacy of zebularine against cholangiocarcinoma cell lines and report here that zebularine has an antitumor effect against cholangiocarcinoma.

We observed that zebularine affected each of the five CCA cell lines examined in this study. There were, however, differences in sensitivity to zebularine among the five cell lines. Further studies will be needed to clarify why this is; regardless of the reason, however, these results suggest that the effect of zebularine as an antitumor reagent against CCA depends on CCA type.

In this study, we demonstrated that zebularine decreased the levels of DNMT3a, DNMT3b, and especially DNMT1 in TFK-1 and HuCCT1 cells. These results were similar to those of other reports showing that DNMT inhibitors deplete DNMT1, 3a, and 3b proteins in human bladder, breast, and cervical cancer cells and hepatocellular carcinoma [30, 31, 38, 47]. In the HeLa cervical cancer cell line, it has been reported that DNMT3a and 3b are the proteins most strongly reduced by zebularine [38]. These results suggest that the major target DNMTs of zebularine depend on the type of cancer cell being targeted. DNMT1 is often referred to as the maintenance methyltransferase, because it is believed to be the enzyme primarily responsible for copying methylation patterns after DNA replication [48]. DNMT3a and DNMT3b, on the other hand, cannot differentiate between unmethylated and hemi-methylated CpG sites and cannot copy a specific pattern of methylation or contribute to the maintenance of methylation patterns [49]. Because they show no preference for hemi-methylated DNA, both enzymes appear to function as *de novo* methyltransferases [49]. The expression levels of these DNMTs are reportedly elevated in several cancers [42–46]. In this study we also observed that DNMT1 was upregulated in CCA tissues compared with normal tissues. The role of altered expression of DNMTs in DNA hypomethylation and hypermethylation in cancer is uncertain and may involve changes in mRNA or protein expression [50]. Inhibition of DNMTs correlates with reduction in tumorigenicity and increased expression of tumor suppressor genes [51]. Hence, DNMTs are considered valuable targets for the design of specific anticancer strategies [50]. The balance of all three enzymes, their accumulative and coordinated effects, and the effects of any inhibitions they may cause must be studied in cancer cells in order to develop effective chemotherapy reagents and to determine the optimal usage of zebularine as a DNA methylation inhibitor drug against each cell type including CCA.

We have previously reported that zebularine exerts antitumor activity on hepatocellular carcinoma HepG2 cells by inhibiting cell proliferation and inducing apoptosis, though it has little effect on DNA methylation in HepG2 cells [31]. Recently, Yang et al. have demonstrated that zebularine exerts anticancer activity against colorectal cancer in cell cultures and in mice through p53-dependent apoptosis, ER stress and autophagy [40]. Yang et al.’s study and our own work have uncovered novel DNMT inhibition-independent action mechanisms of zebularine and have shed new light on zebularine’s potential as a treatment for clinical colorectal cancer and hepatocellular cancer, respectively. On the other hand, previous works have focused on elucidating the molecular mechanism responsible for zebularine’s function as a DNA methylation inhibitor [29]. In this study, we also demonstrated that zebularine treatment decreased DNMT levels and that DNMT1 knockdown led to decreased cell viability in TFK-1 and HuCCT1 cells. In addition, we demonstrated that zebularine treatment altered DNA methylation status and that zebularine reverses the promoter hypermethylation of protocadherin, transcriptional regulator, and Wnt signaling-related genes in TFK-1 and HuCCT1 cells. These results indicate that zebularine functions through DNMT inhibition-dependent action mechanisms in TFK-1 and HuCCT1 cells as an anticancer reagent. Thus, zebularine has potential as an anticancer compound as both a DNA methylation inhibitor and a non-DNA methylation inhibitor, and can function in either role depending on cell type.

In this study, we demonstrate that zebularine inhibits TFK-1 and HuCCT1 cell growth by inducing apoptosis. During the process of apoptosis, caspases are essential for the initiation and execution of cell death in a self-amplifying cascade in response to various stimuli [52]. Two major apoptotic pathways have been identified: the extrinsic and intrinsic apoptotic pathways. The extrinsic pathway is activated by death receptors, which recruit initiator caspase-2, -8, or-10 through adaptor molecules, whereas the intrinsic signals activate caspase-9. These initiator caspases can sequentially cleave and activate the effector caspases (caspase-3, -6, and -7), which play important roles in mediating cellular destruction [53]. Our results showed that zebularine appeared to induce the apoptosis of CCA via the intrinsic pathway, as shown by the activation of caspase-9, and also via the extrinsic pathway, as shown by the activation of caspase-8 followed by caspase-3.

At the same time, our results also showed that DNA replication was blocked by treatment with zebularine in HuCCT1 cells, though not in TFK-1 cells. This suggests that there are some differences among CCA types in terms of the effects of zebularine. Eukaryotic cell proliferation is a highly regulated system that is controlled by CDK-cyclin complexes. The cell-cycle transition from the G1 to the S phase is the major regulatory checkpoint in this process. This transition is characterized by the phosphorylation of Rb and catalyzed by the CDK-cyclin complex [54, 55]. In this study, we could not detect any alteration of cell cycle regulation arising from zebularine treatment. The action mechanism of zebularine for HuCCT1 cell cycle regulation must be studied to enable us to develop optimal chemotherapy for CAA.

In the present study, we demonstrated that zebularine treatments induce DNA demethylation of Wnt signaling pathway-related genes and decrease β-catenin protein levels in CCA cells. The Wnt signaling pathway regulates cell proliferation, differentiation, apoptosis and other biological processes. The activation of the Wnt signaling pathway is closely related to tumorigenesis and progression in various types of tumors [56, 57]. Aberrant activation of the Wnt signaling pathway is known to be closely related to one subtype of CCA [58]. β-catenin gene mutations have also been detected in a few patients with CCA, suggesting that tyrosine phosphorylation-dependent β-catenin activation or Wnt/Frizzled dysfunction may contribute to the genesis of CCA [59]. A previous study demonstrated that the Wnt signaling pathway is activated in CCA cell lines and that blocking the Wnt signaling pathway enhances cell apoptosis and suppresses cell proliferation [60]. Thirty-one genes categorized as “Wnt signaling pathway” genes demethylated due to zebularine treatment in this study, including both Wnt signaling enhancer genes and the repressor gene. For instance, *SFRP1* belongs to the secreted glycoprotein SFRP family (SFRP1 to 5), the members of which are antagonists of Wnt signaling pathways [61]. These results suggest that zebularine eventually suppresses Wnt signaling via DNA demethylation of Wnt signaling pathway-related genes and thereby exerts an antitumor effect against CCA cell lines.

It must be considered, however, that a number of previous studies have suggested that zebularine has limited bioavailability in humans. One of these showed that zebularine’s DNA methyltransferase inhibition effect is reversible, and also that tumors must be exposed to zebularine for long periods for inhibition of tumor growth. Holleran et al. pointed out that, given the terminal half-life of zebularine after intravenous injection into mice, rats, and monkeys, it is likely that frequent dosing or continuous intravenous infusion of zebularine will be necessary to maintain prolonged inhibition of DNA methyltransferase. Although oral administration of zebularine has been shown to have *in-vivo* activity in preclinical murine tumor models, and oral administration of zebularine would be the most logistically expedient means of delivering regular and relatively frequent doses of zebularine, the low bioavailability of zebularine in rodents and the extremely low bioavailability of zebularine in rhesus monkeys are a cause for concern [62]. This is one of the reasons why zebularine has not been further developed as a form of chemotherapy. Since zebularine has anticancer effects against many type of tumors, however, it remains promising, and further investigation of the various antitumor action mechanisms of zebularine may reveal a way in which it can be used as a novel chemotherapeutic strategy against cancer.

The present study does not include an animal model of CCA, which will be an important step in proving that zebularine treatment can be effective against CCA in practice. In order to investigate the *in-vivo* anti-CCA activity of zebularine, the action and mechanisms of zebularine must be further investigated in animal models such as xenograft and orthotopic models, carcinogen-induced CCA models, and genetically engineered mouse models of CCA.

## Conclusion

Our observations indicate that zebularine induces apoptotic cell death in CCA cell lines. Its effects are dependent on DNMT1 depletion and DNA demethylation, which induces the activation of certain genes including protocadherin, transcriptional regulators, and Wnt signaling-related genes. Zebularine also inhibits cell growth in certain CCA cell lines; this may contribute to its antiproliferation effects against some types of CCA cells. Our present and previous (31) reports have shown that zebularine could function as both a DNMT inhibitor and a non-DNMT inhibitor reagent, and that, when administered in the optimal manner for each cell type, zebularine may be useful for chemotherapy against cancer.

## Supporting Information

S1 Fig Effect of zebularine on cell viability and DNMT expression in CCA cell lines. TFK-1 (A), HuCCT1 (B), KKU-100 (C), KKU-M156 (D) and KKU-M213 (E) cells were treated with zebularine at indicated concentrations for 72 h. Cell growth was measured by CellTiter-Glo Luminescent Cell Viability Assay. Data are the means ± SEM of results from at least three independent experiments. *p < 0.05, compared to 0 μM.
(TIF)

S2 Fig The map 04310 for "Wnt signaling pathway" in the Kyoto Encyclopedia of Genes and Genomes (KEGG) Database. The 31 genes included among the 2,102 genes hosting 3,309 hypomethylated CpG sites (see S2 Table and the main text for details) are marked by red stars.
(PPTX)

S3 Fig Visualization of the beta and delta-beta values of TFK1 and HuCCT1 cell lines after zebularine treatment. Visualization of the beta and delta-beta values of TFK1 and HuCCT1 cell lines after zebularine treatment together with the beta values of human normal tissues (from GEO accession numbers GSE52578 and GSE30870) for protocadherin gene cluster (A), HOXA gene cluster (B), homeobox genes (IRX2 and TLX3 as examples) (C, D), and Wnt signaling-related genes (E-J) using the Integrative Genomics Viewer (IGV, www.broadinstitute.org/igv/home). The data range shown is 0 to 1 for β values and -0.5 to 0.5 for delta-beta (Δ-β) values.
(PPTX)

S1 Table List of 4,285 CpG sites that were hypomethylated (delta-beta < -0.2) in zebularine-treated TFK1 and HuCCT1 cells. (XLS)

S2 Table The results of gene ontology term analysis using DAVID for the 2,102 genes hosting 3,309 CpG sites. The results of the gene ontology term analysis using DAVID for the 2,102 genes hosting 3,309 CpG sites commonly hypomethylated (delta-beta < -0.2) in TFK1 and HuCCT1 cells after zebularine (1000μM) treatment.
(XLS)

S3 Table The results of gene ontology term analysis using DAVID for the 782 genes hosting 948 CpG sites. The results of the gene ontology term analysis using DAVID for the 782 genes hosting 948 CpG sites that are located in the pTSS regions and are commonly hypomethylated (delta-beta < -0.2) after zebularine (1000μM) treatment in TFK1 and HuCCT1 cells.
(XLS)

S4 Table List of 31 genes categorized in "Wnt signaling pathway" and included among the 2,102 genes hosting 3,309 hypomethylated CpG sites. (XLS)
