# Supplementary figures and images for: DNA Methyltransferase Inhibitor Zebularine Induces Human Cholangiocarcinoma Cell Death through Alteration of DNA Methylation Status
Source: PLoS One. 2015 Mar 23;10(3):e0120545. doi: 10.1371/journal.pone.0120545 (PMC4370694; doi:10.1371/journal.pone.0120545)

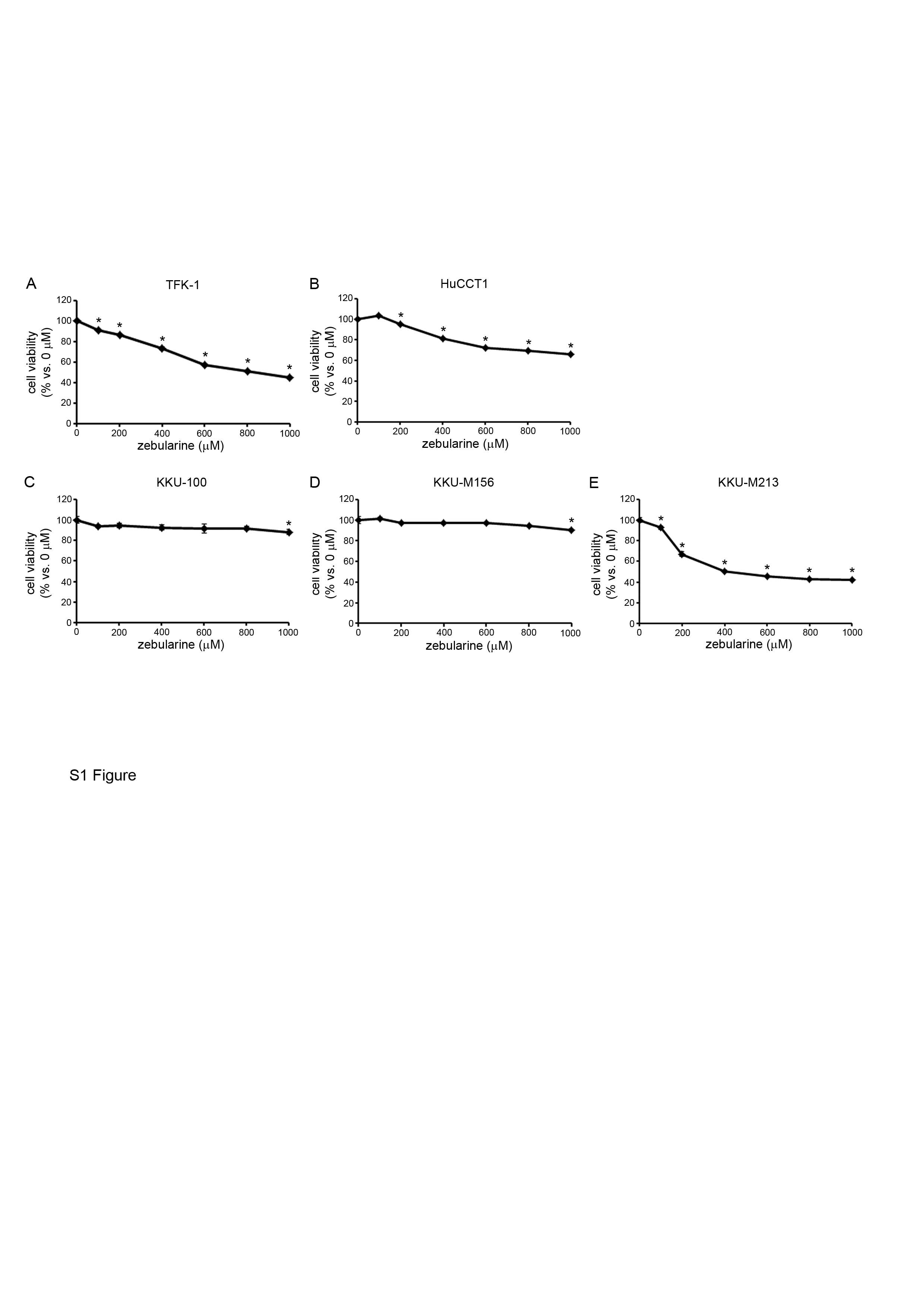

Supplement: S1 Fig — TFK-1 (A), HuCCT1 (B), KKU-100 (C), KKU-M156 (D) and KKU-M213 (E) cells were treated with zebularine at indicated concentrations for 72 h. Cell growth was measured by CellTiter-Glo Luminescent Cell Viability Assay. Data are the means ± SEM of results from at least three independent experiments. *p < 0.05, compared to 0 μM. (TIF) [file pone.0120545.s001.tif]
