# Supplementary material for: DNA Methyltransferase Inhibitor Zebularine Induces Human Cholangiocarcinoma Cell Death through Alteration of DNA Methylation Status
Source: PLoS One. 2015 Mar 23;10(3):e0120545. doi: 10.1371/journal.pone.0120545 (PMC4370694; doi:10.1371/journal.pone.0120545)
Supplement: S2 Fig — The 31 genes included among the 2,102 genes hosting 3,309 hypomethylated CpG sites (see S2 Table and the main text for details) are marked by red stars. (PPTX) [file pone.0120545.s002.pptx]

## Slide 1
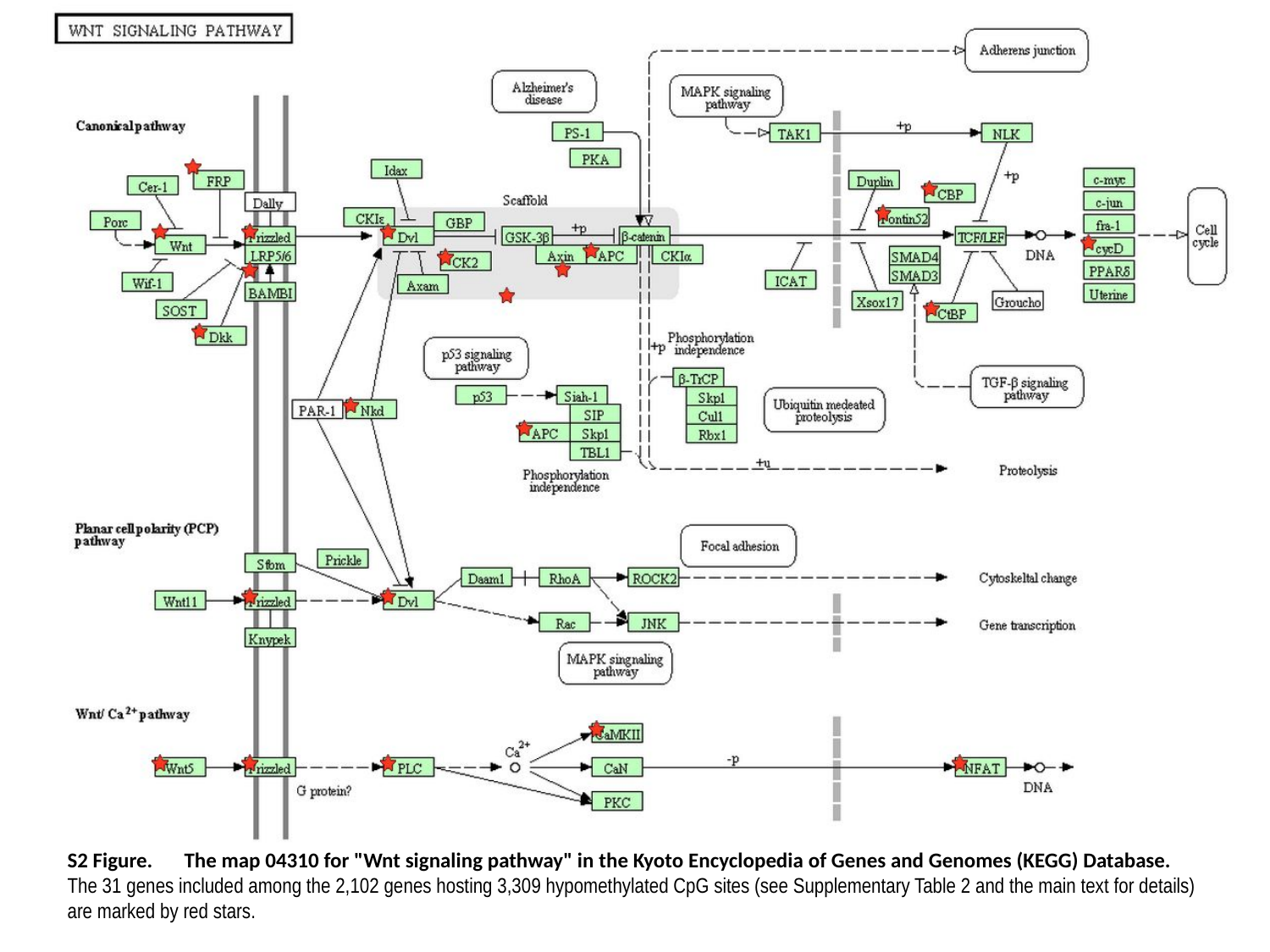

S2 Figure.　The map 04310 for "Wnt signaling pathway" in the Kyoto Encyclopedia of Genes and Genomes (KEGG) Database.
The 31 genes included among the 2,102 genes hosting 3,309 hypomethylated CpG sites (see Supplementary Table 2 and the main text for details)
are marked by red stars.
