# Supplementary material for: DNA Methyltransferase Inhibitor Zebularine Induces Human Cholangiocarcinoma Cell Death through Alteration of DNA Methylation Status
Source: PLoS One. 2015 Mar 23;10(3):e0120545. doi: 10.1371/journal.pone.0120545 (PMC4370694; doi:10.1371/journal.pone.0120545)
Supplement: S3 Fig — Visualization of the beta and delta-beta values of TFK1 and HuCCT1 cell lines after zebularine treatment together with the beta values of human normal tissues (from GEO accession numbers GSE52578 and GSE30870) for protocadherin gene cluster (A), HOXA gene cluster (B), homeobox genes (IRX2 and TLX3 as examples) (C, D), and Wnt signaling-related genes (E-J) using the Integrative Genomics Viewer (IGV, www.broadinstitute.org/igv/home). The data range shown is 0 to 1 for β values and -0.5 to 0.5 for delta-beta (Δ-β) values. (PPTX) [file pone.0120545.s003.pptx]

## Slide 1
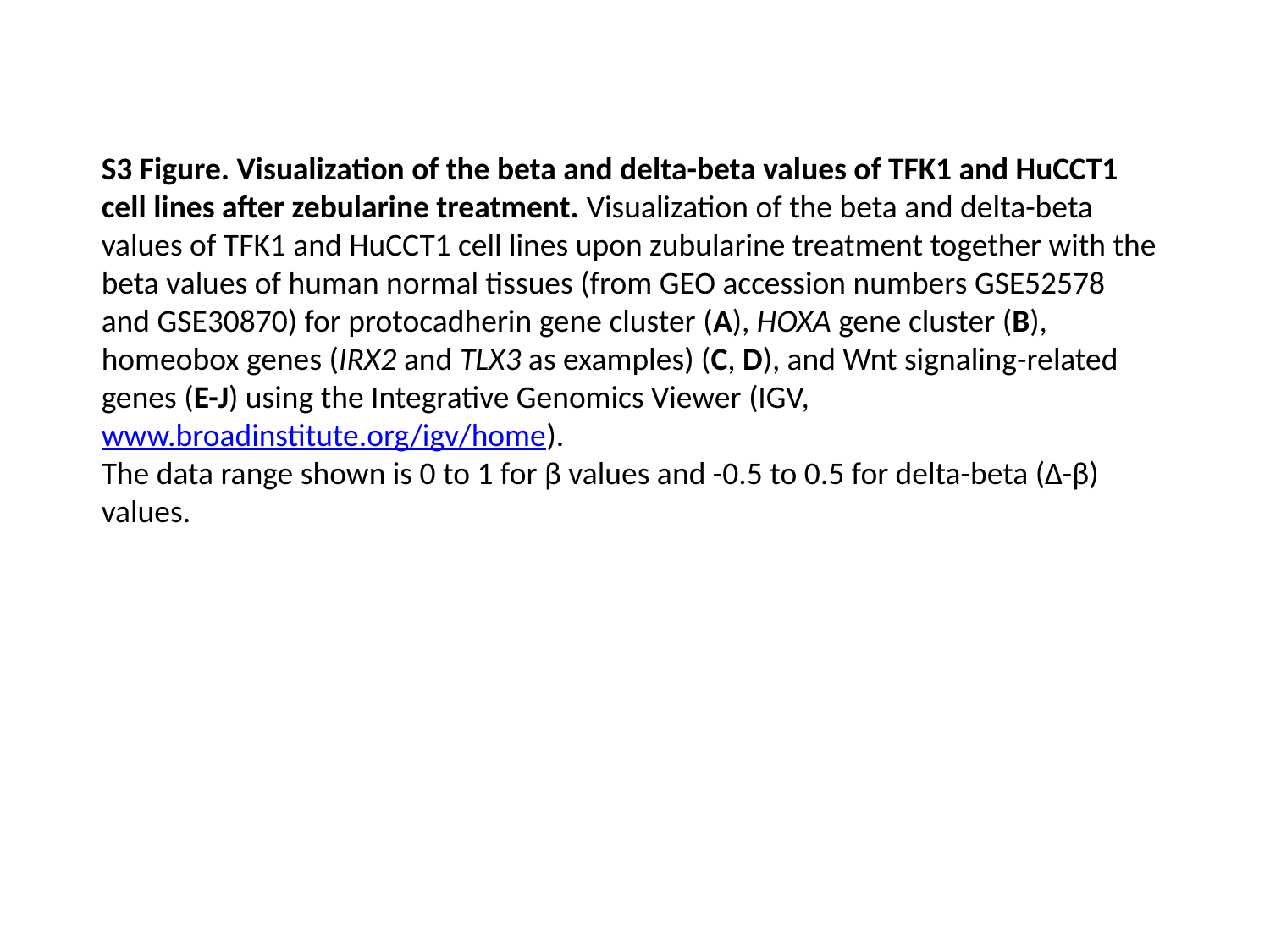

S3 Figure. Visualization of the beta and delta-beta values of TFK1 and HuCCT1 cell lines after zebularine treatment. Visualization of the beta and delta-beta values of TFK1 and HuCCT1 cell lines upon zubularine treatment together with the beta values of human normal tissues (from GEO accession numbers GSE52578 and GSE30870) for protocadherin gene cluster (A), HOXA gene cluster (B), homeobox genes (IRX2 and TLX3 as examples) (C, D), and Wnt signaling-related genes (E-J) using the Integrative Genomics Viewer (IGV, www.broadinstitute.org/igv/home).
The data range shown is 0 to 1 for β values and -0.5 to 0.5 for delta-beta (Δ-β) values.

## Slide 2
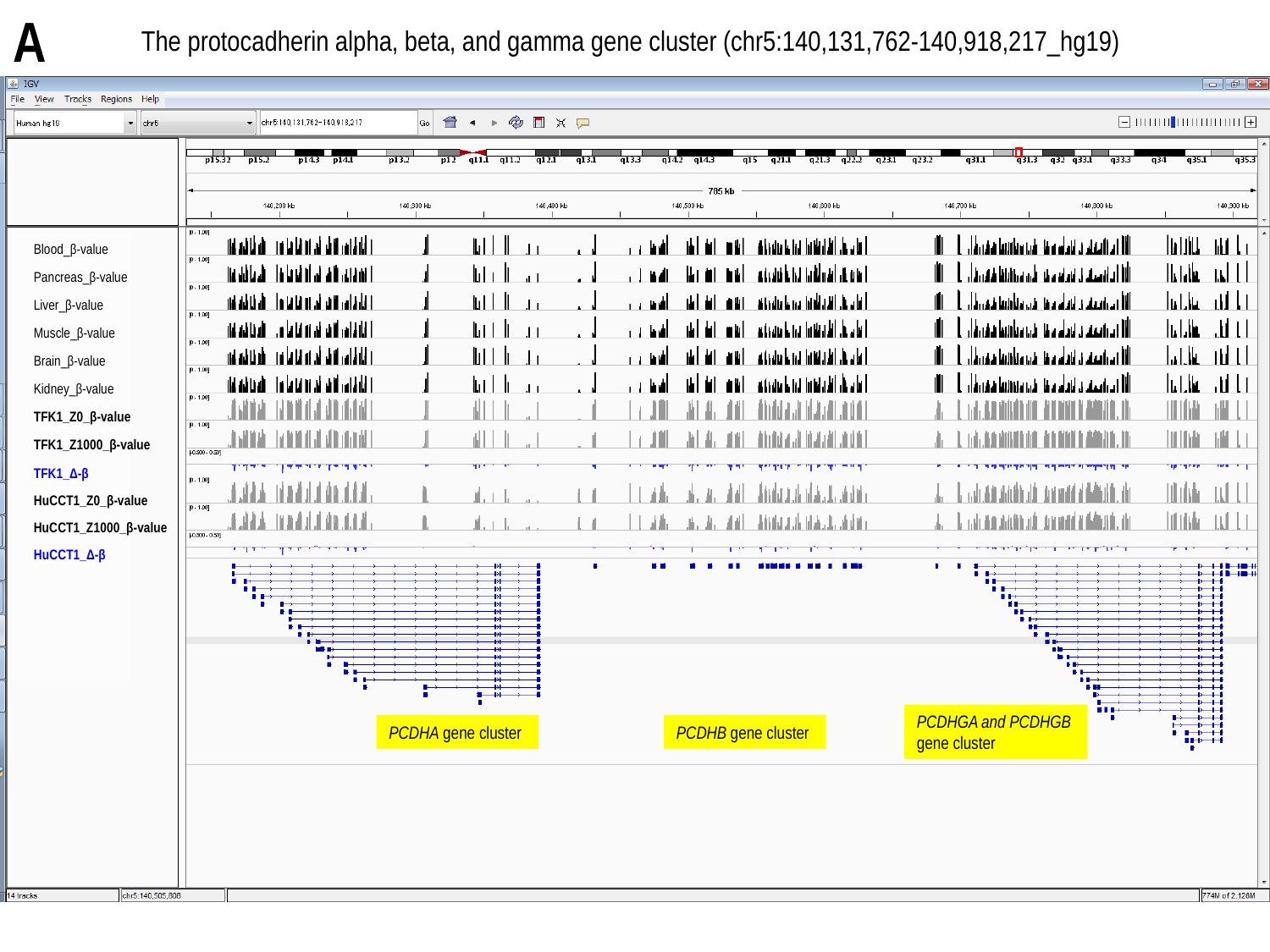

A
Blood_β-value
Pancreas_β-value
Liver_β-value
Muscle_β-value
Brain_β-value
TFK1_Z0_β-value
TFK1_Z1000_β-value
TFK1_Δ-β
HuCCT1_Z0_β-value
HuCCT1_Z1000_β-value
HuCCT1_Δ-β
The protocadherin alpha, beta, and gamma gene cluster (chr5:140,131,762-140,918,217_hg19)
Blood_β-value
Pancreas_β-value
Liver_β-value
Muscle_β-value
Brain_β-value
Kidney_β-value
TFK1_Z0_β-value
TFK1_Z1000_β-value
TFK1_Δ-β
HuCCT1_Z0_β-value
HuCCT1_Z1000_β-value
HuCCT1_Δ-β
PCDHGA and PCDHGB
gene cluster
PCDHA gene cluster
PCDHB gene cluster

## Slide 3
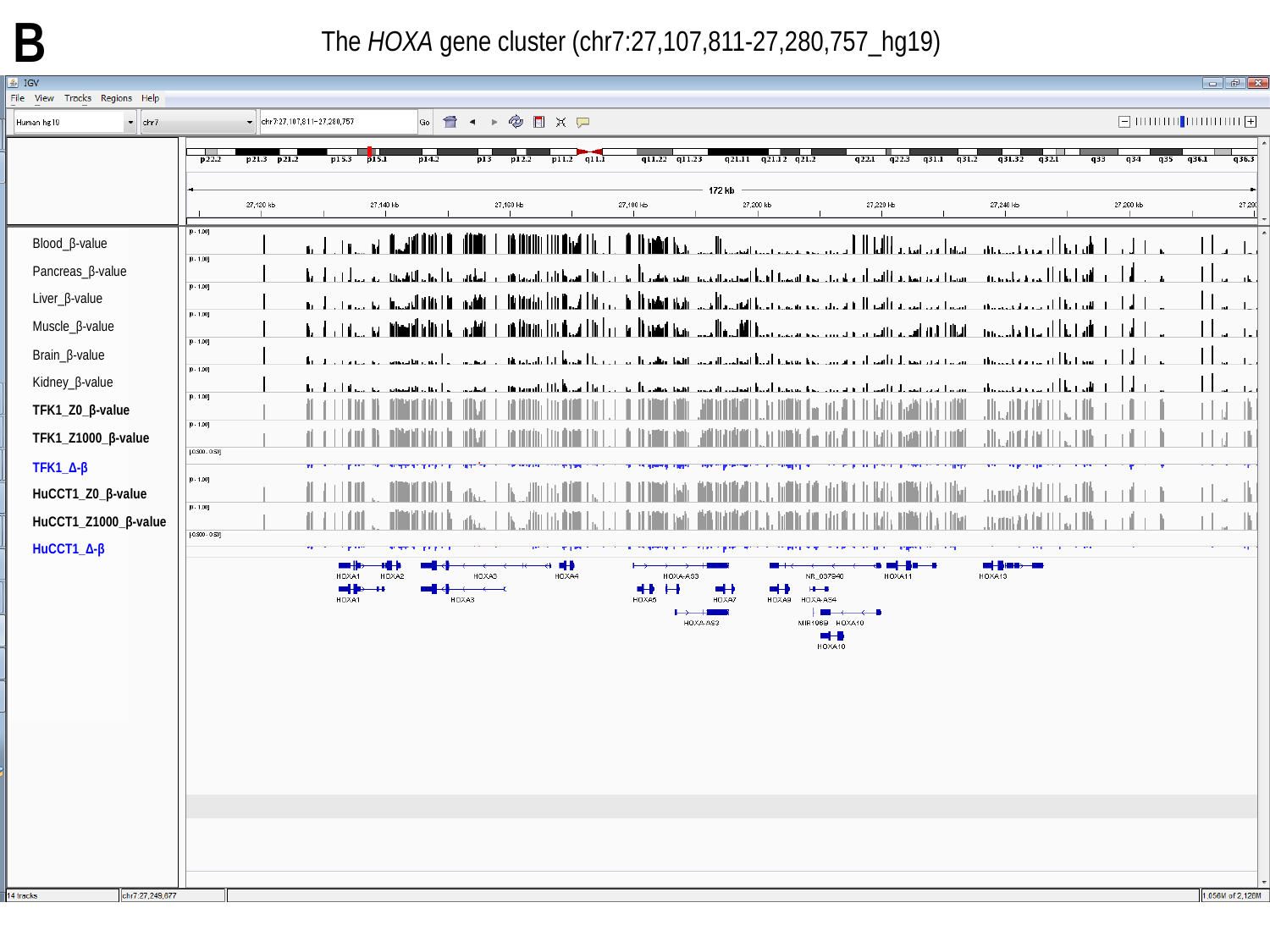

B
The HOXA gene cluster (chr7:27,107,811-27,280,757_hg19)
Blood_β-value
Pancreas_β-value
Liver_β-value
Muscle_β-value
Brain_β-value
Kidney_β-value
TFK1_Z0_β-value
TFK1_Z1000_β-value
TFK1_Δ-β
HuCCT1_Z0_β-value
HuCCT1_Z1000_β-value
HuCCT1_Δ-β

## Slide 4
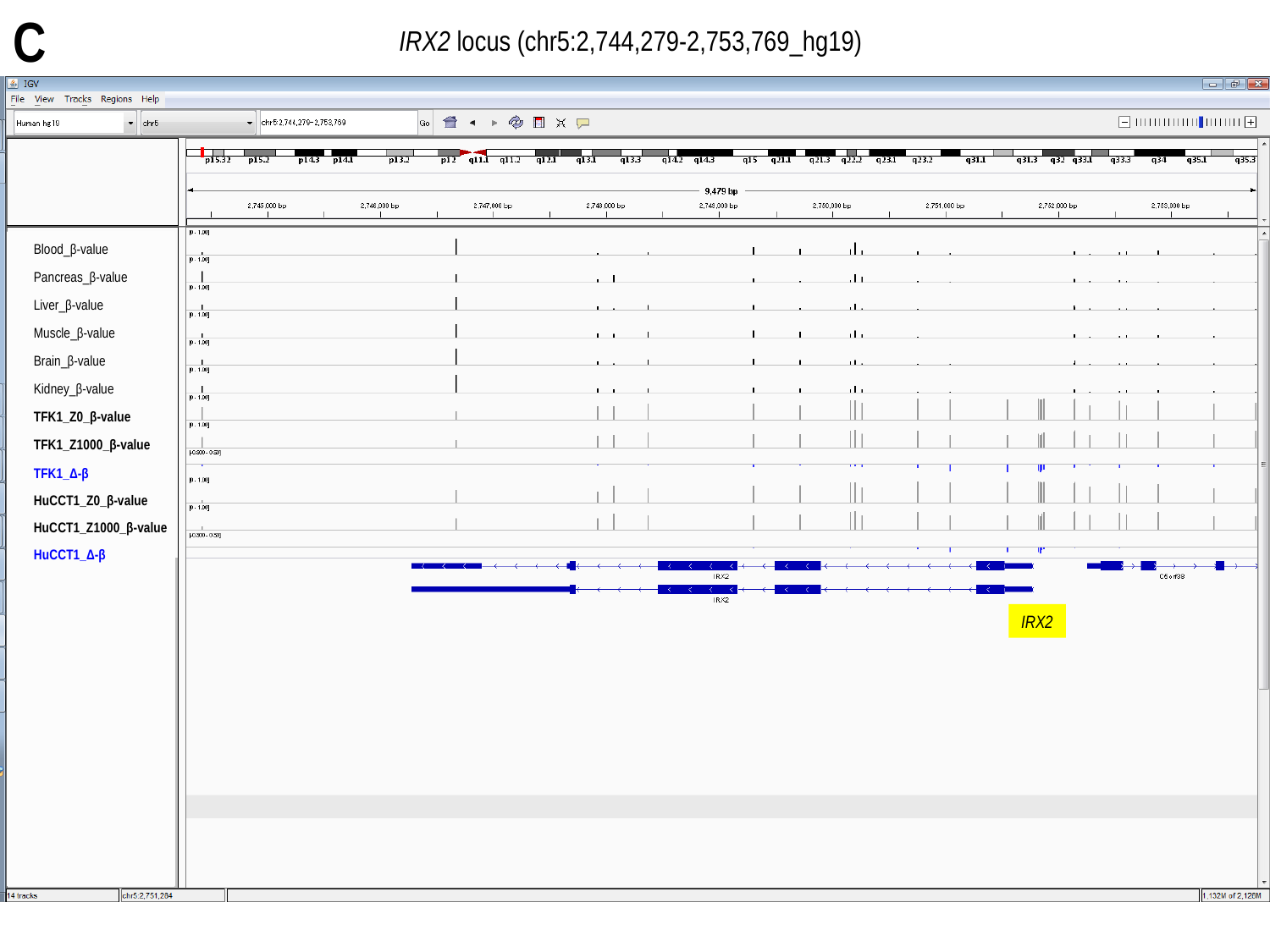

C
IRX2 locus (chr5:2,744,279-2,753,769_hg19)
Blood_β-value
Pancreas_β-value
Liver_β-value
Muscle_β-value
Brain_β-value
Kidney_β-value
TFK1_Z0_β-value
TFK1_Z1000_β-value
TFK1_Δ-β
HuCCT1_Z0_β-value
HuCCT1_Z1000_β-value
HuCCT1_Δ-β
IRX2

## Slide 5
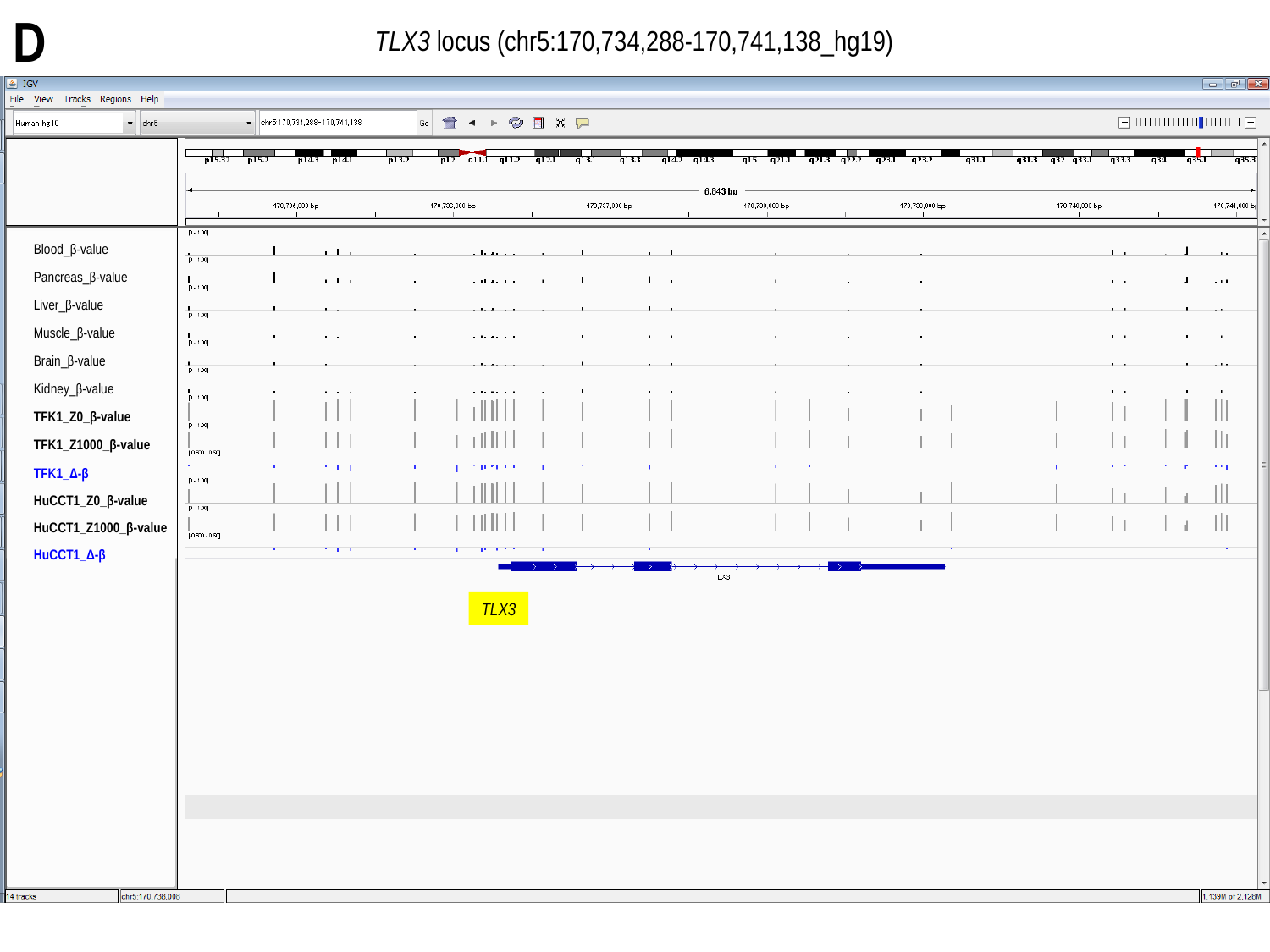

D
TLX3 locus (chr5:170,734,288-170,741,138_hg19)
Blood_β-value
Pancreas_β-value
Liver_β-value
Muscle_β-value
Brain_β-value
Kidney_β-value
TFK1_Z0_β-value
TFK1_Z1000_β-value
TFK1_Δ-β
HuCCT1_Z0_β-value
HuCCT1_Z1000_β-value
HuCCT1_Δ-β
TLX3

## Slide 6
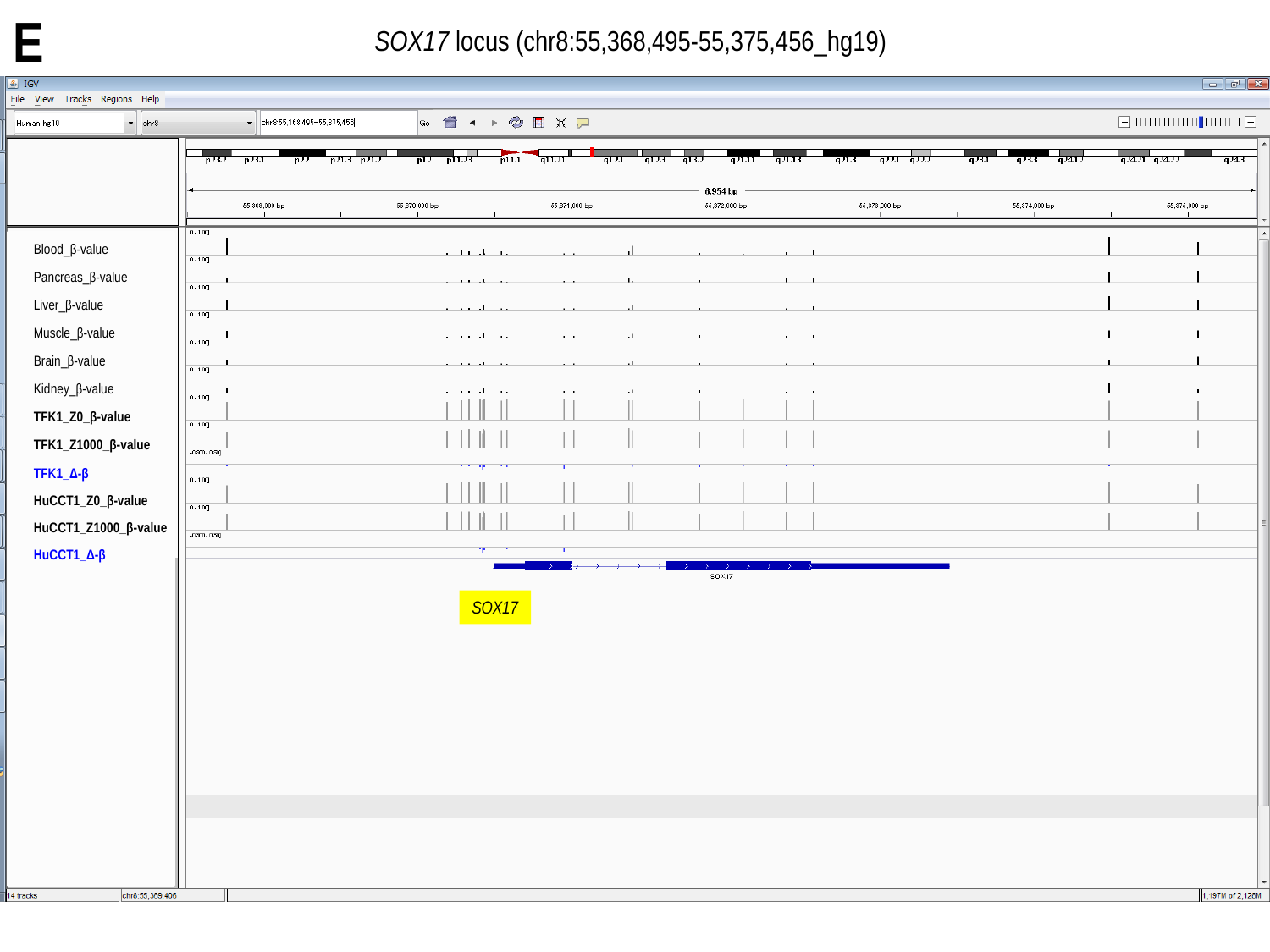

E
SOX17 locus (chr8:55,368,495-55,375,456_hg19)
Blood_β-value
Pancreas_β-value
Liver_β-value
Muscle_β-value
Brain_β-value
Kidney_β-value
TFK1_Z0_β-value
TFK1_Z1000_β-value
TFK1_Δ-β
HuCCT1_Z0_β-value
HuCCT1_Z1000_β-value
HuCCT1_Δ-β
SOX17

## Slide 7
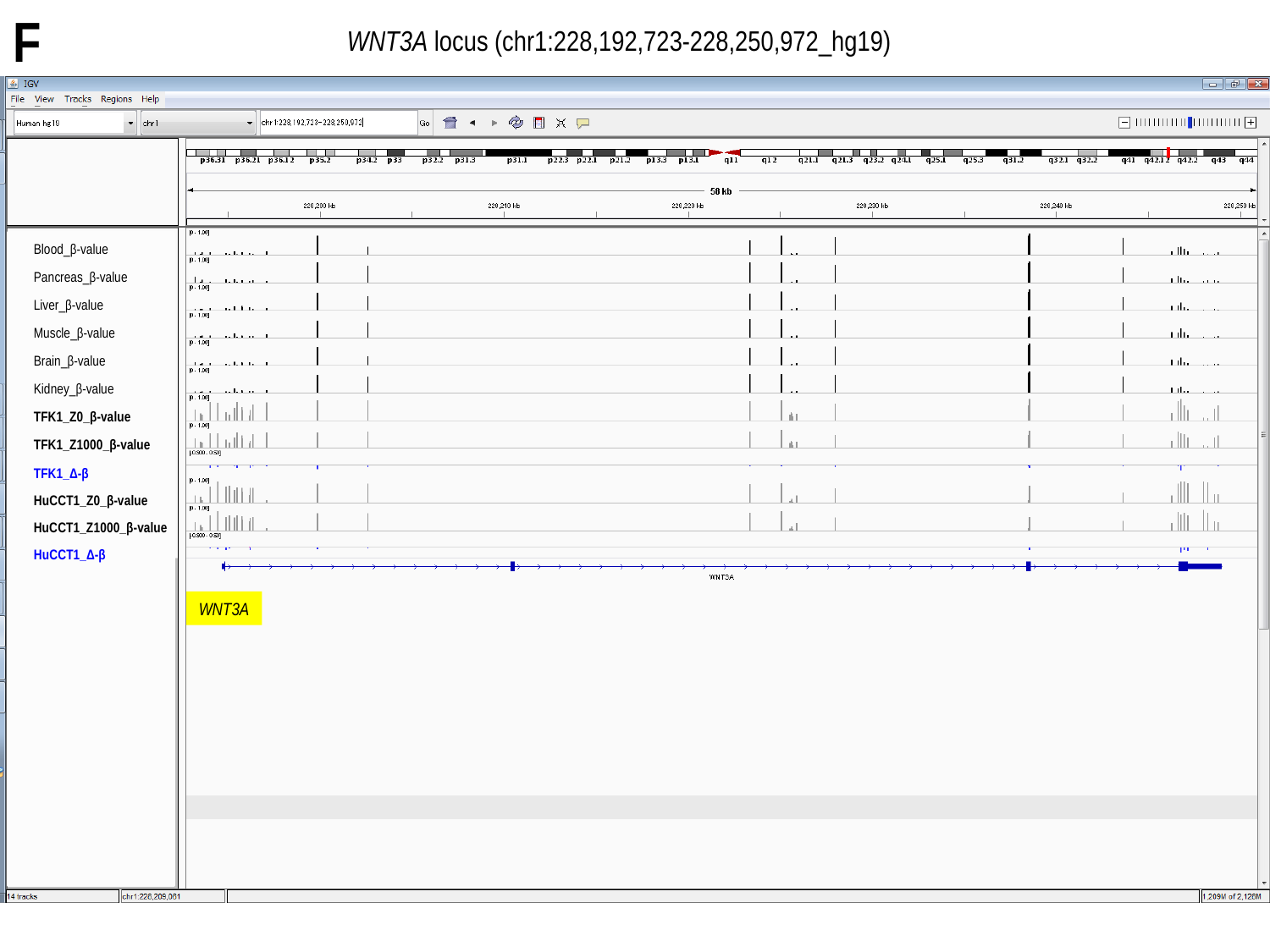

F
WNT3A locus (chr1:228,192,723-228,250,972_hg19)
Blood_β-value
Pancreas_β-value
Liver_β-value
Muscle_β-value
Brain_β-value
Kidney_β-value
TFK1_Z0_β-value
TFK1_Z1000_β-value
TFK1_Δ-β
HuCCT1_Z0_β-value
HuCCT1_Z1000_β-value
HuCCT1_Δ-β
WNT3A

## Slide 8
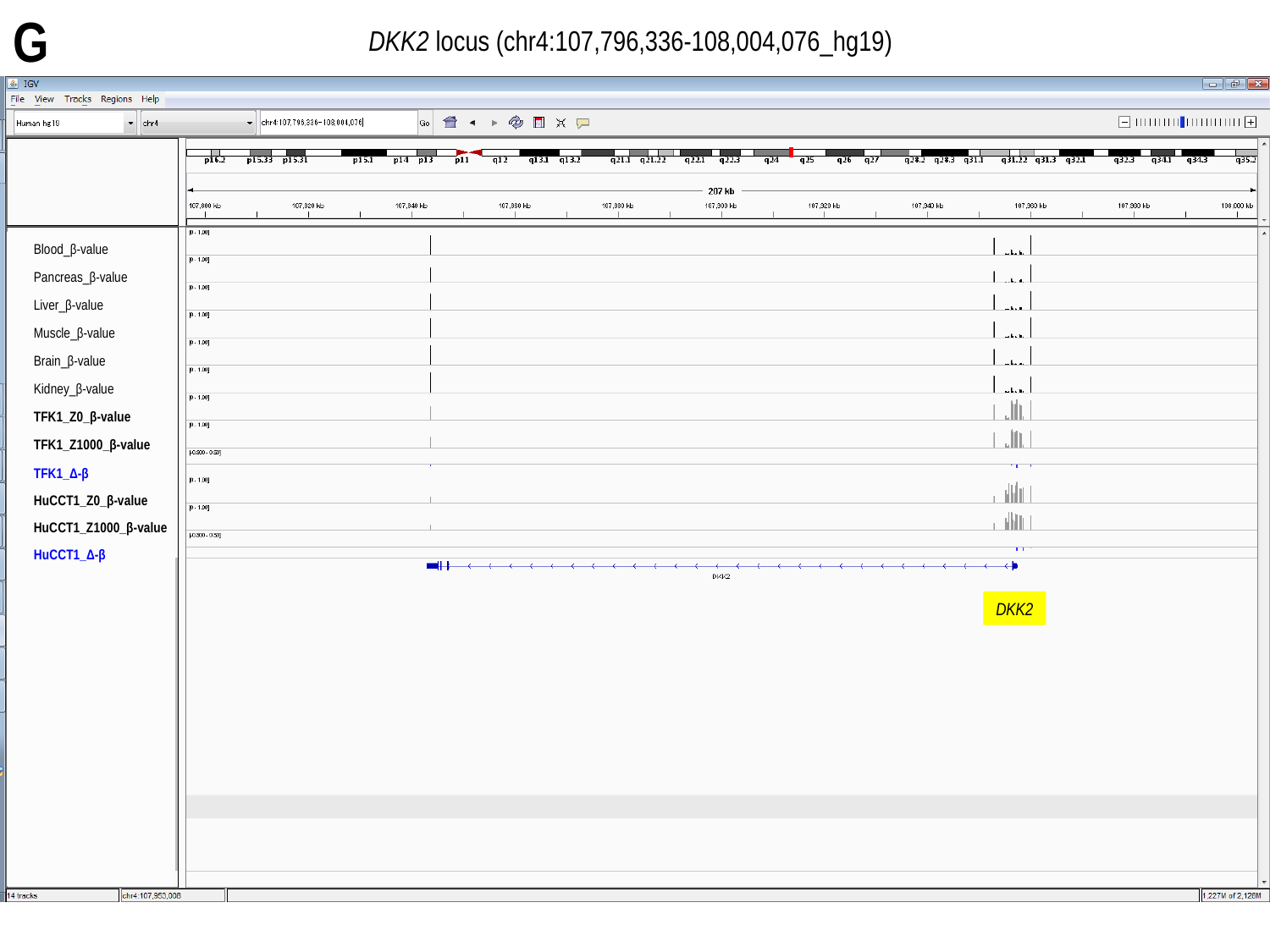

G
DKK2 locus (chr4:107,796,336-108,004,076_hg19)
Blood_β-value
Pancreas_β-value
Liver_β-value
Muscle_β-value
Brain_β-value
Kidney_β-value
TFK1_Z0_β-value
TFK1_Z1000_β-value
TFK1_Δ-β
HuCCT1_Z0_β-value
HuCCT1_Z1000_β-value
HuCCT1_Δ-β
DKK2

## Slide 9
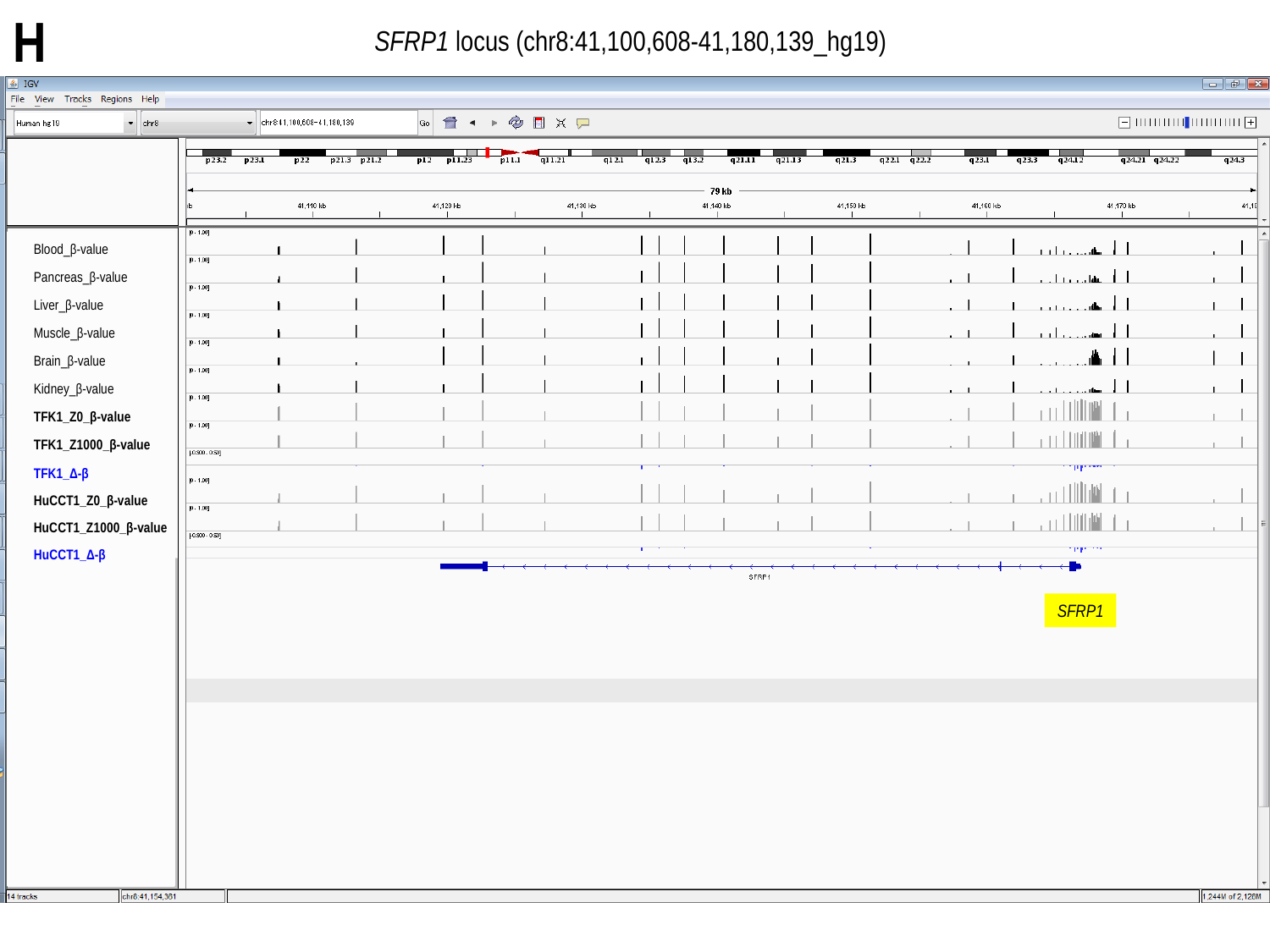

H
SFRP1 locus (chr8:41,100,608-41,180,139_hg19)
Blood_β-value
Pancreas_β-value
Liver_β-value
Muscle_β-value
Brain_β-value
Kidney_β-value
TFK1_Z0_β-value
TFK1_Z1000_β-value
TFK1_Δ-β
HuCCT1_Z0_β-value
HuCCT1_Z1000_β-value
HuCCT1_Δ-β
SFRP1

## Slide 10
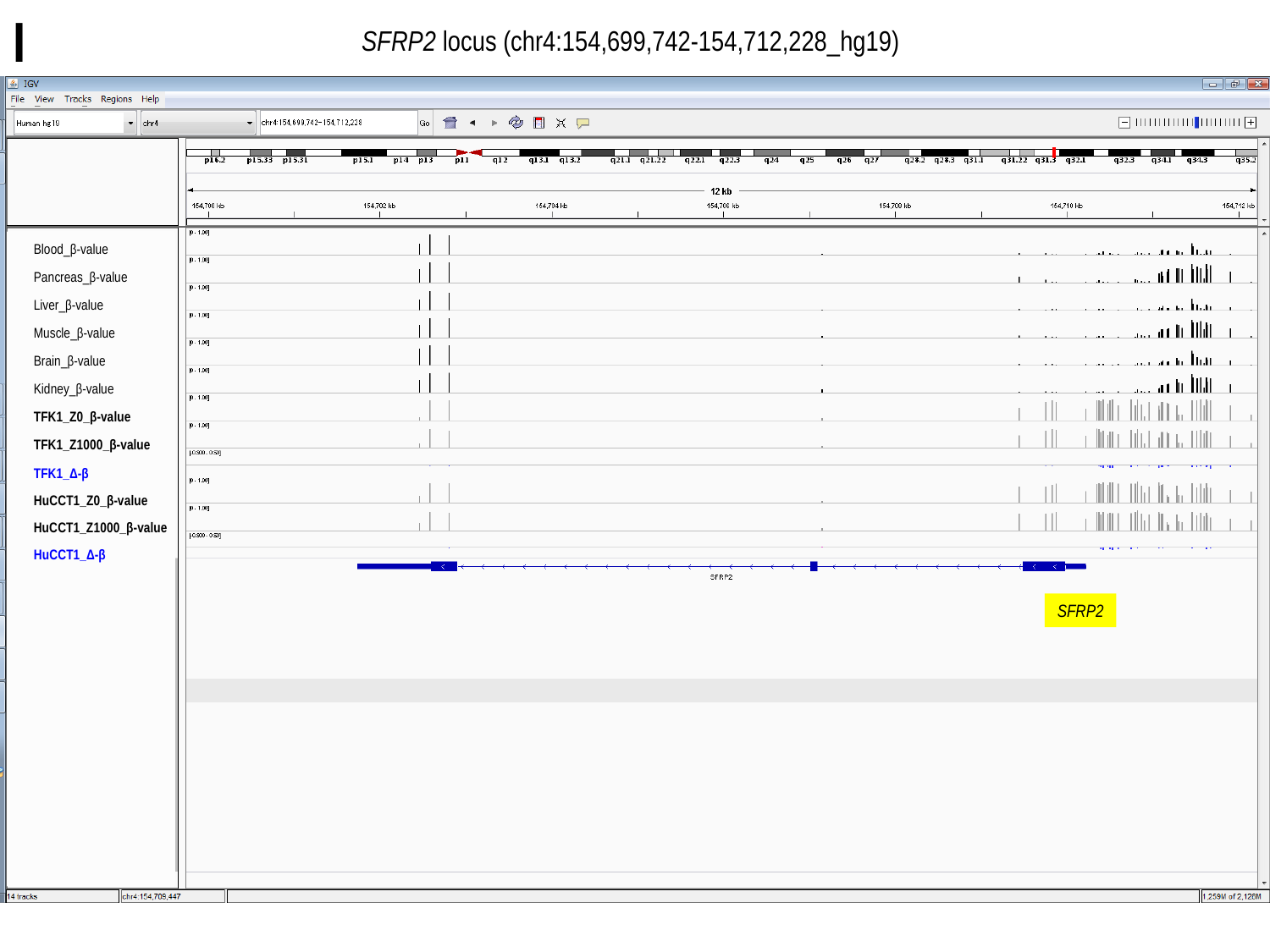

I
SFRP2 locus (chr4:154,699,742-154,712,228_hg19)
Blood_β-value
Pancreas_β-value
Liver_β-value
Muscle_β-value
Brain_β-value
Kidney_β-value
TFK1_Z0_β-value
TFK1_Z1000_β-value
TFK1_Δ-β
HuCCT1_Z0_β-value
HuCCT1_Z1000_β-value
HuCCT1_Δ-β
SFRP2

## Slide 11
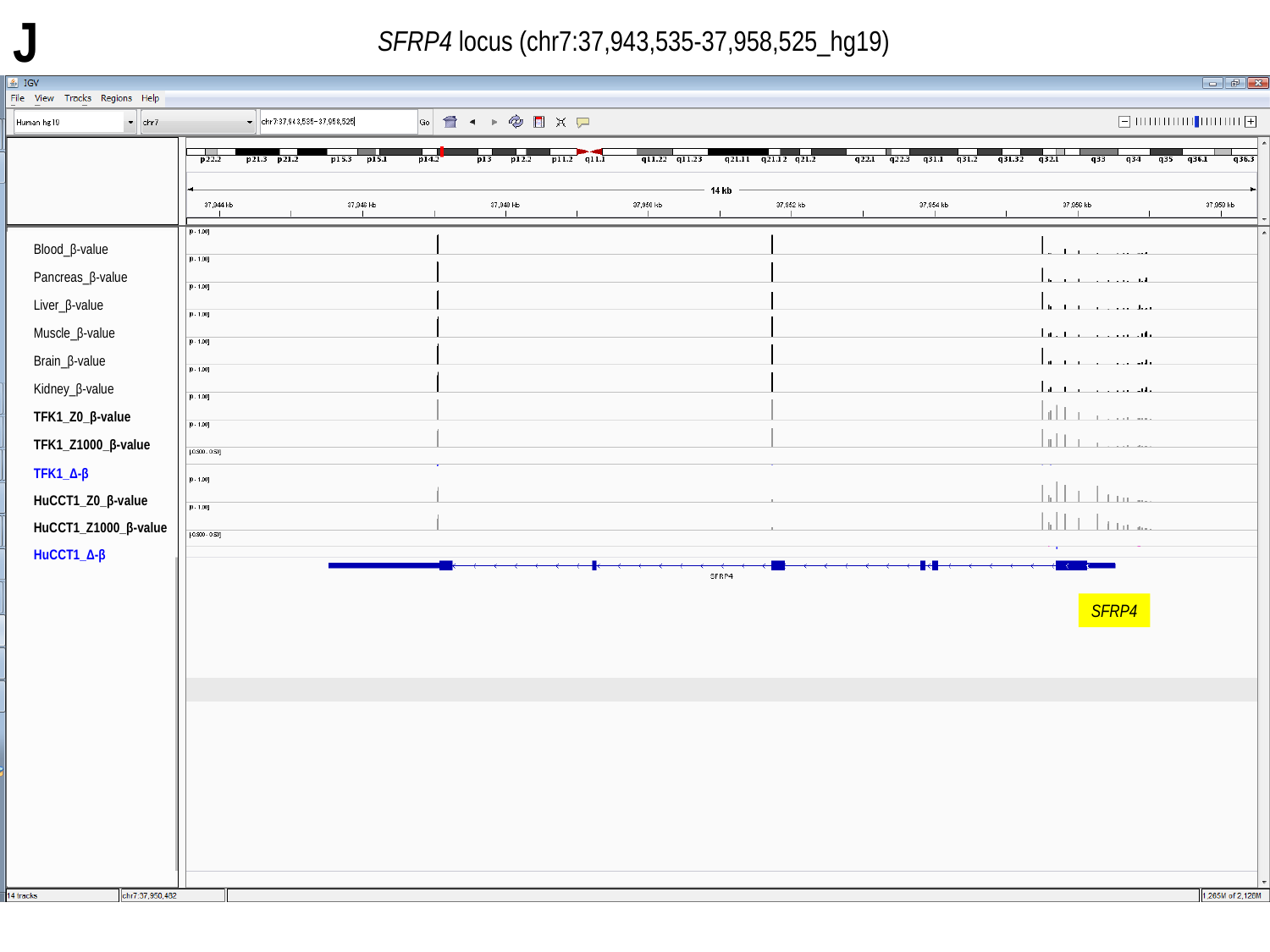

J
SFRP4 locus (chr7:37,943,535-37,958,525_hg19)
Blood_β-value
Pancreas_β-value
Liver_β-value
Muscle_β-value
Brain_β-value
Kidney_β-value
TFK1_Z0_β-value
TFK1_Z1000_β-value
TFK1_Δ-β
HuCCT1_Z0_β-value
HuCCT1_Z1000_β-value
HuCCT1_Δ-β
SFRP4
